# Penalized regression models to select biomarkers of environmental enteric dysfunction associated with linear growth acquisition in a Peruvian birth cohort

**DOI:** 10.1371/journal.pntd.0007851

**Published:** 2019-11-15

**Authors:** Josh M. Colston, Pablo Peñataro Yori, Lawrence H. Moulton, Maribel Paredes Olortegui, Peter S. Kosek, Dixner Rengifo Trigoso, Mery Siguas Salas, Francesca Schiaffino, Ruthly François, Fahmina Fardus-Reid, Jonathan R. Swann, Margaret N. Kosek

**Affiliations:** 1 Department of International Health, Johns Hopkins Bloomberg School of Public Health, Baltimore, Maryland, United States of America; 2 Division of Infectious Diseases and International Health, University of Virginia School of Medicine, Charlottesville, Virginia, United States of America; 3 Department of International Health, Johns Hopkins School of Public Health, Baltimore, Maryland, United States of America; 4 Research Unit, Asociación Benéfica Prisma, Iquitos, Peru; 5 Oregon Neurosurgery, Eugene, Oregon, United States of America; 6 Division of Integrative Systems Medicine and Digestive Diseases, Department of Surgery and Cancer, Imperial College London, London, United Kingdom; Johns Hopkins Bloomberg School of Public Health, UNITED STATES

## Abstract

Environmental enteric dysfunction (EED) is associated with chronic undernutrition. Efforts to identify minimally invasive biomarkers of EED reveal an expanding number of candidate analytes. An analytic strategy is reported to select among candidate biomarkers and systematically express the strength of each marker’s association with linear growth in infancy and early childhood. 180 analytes were quantified in fecal, urine and plasma samples taken at 7, 15 and 24 months of age from 258 subjects in a birth cohort in Peru. Treating the subjects’ length-for-age Z-score (LAZ-score) over a 2-month lag as the outcome, penalized linear regression models with different shrinkage methods were fitted to determine the best-fitting subset. These were then included with covariates in linear regression models to obtain estimates of each biomarker’s adjusted effect on growth. Transferrin had the largest and most statistically significant adjusted effect on short-term linear growth as measured by LAZ-score–a coefficient value of 0.50 (0.24, 0.75) for each log_2_ increase in plasma transferrin concentration. Other biomarkers with large effect size estimates included adiponectin, arginine, growth hormone, proline and serum amyloid P-component. The selected subset explained up to 23.0% of the variability in LAZ-score. Penalized regression modeling approaches can be used to select subsets from large panels of candidate biomarkers of EED. There is a need to systematically express the strength of association of biomarkers with linear growth or other outcomes to compare results across studies.

## Author summary

Childhood undernutrition is widespread throughout the world and has severe, long-lasting health impacts. Substances measured in blood, urine and stool could be used as biomarkers to identify children undergoing growth failure before these impacts occur. However, it is not yet known which of the many markers that can be identified are accurate and clinically useful predictors of poor growth in infants and children. This study used a large number of candidate biomarkers of immune activation, metabolism and hormones and applied statistical methods to narrow them down from 110 different substances, to the 36 best predictors of growth in 258 Peruvian infants. It also estimated how large the effect of each of these markers was on height two months later. The biomarker with the largest effect was transferrin, a glycoprotein that can be measured in blood samples. 15-month old children with elevated transferrin were around two thirds of a centimeter taller on average at 17 months than those with low levels. Transferrin and other proteins, glycoproteins, hormones and antibodies that this study identified, can be measured easily and affordably in standard laboratories making them feasible to be used broadly as prognostic markers as part of child health and nutrition programs in under-resourced settings.

## Introduction

Chronic undernutrition affects around one in three children under age five, rendering them susceptible to prolonged and more severe infections and putting them at increased risk of mortality [1]. Growth faltering in undernourished children begins to accrue early in life, is generally irreversible and leads to chronic sequelae such as impaired cognitive development and short stature that last into adulthood impeding economic productivity and increasing the risk of low birthweight in offspring [2]. Many evidence-based interventions targeting infant growth demonstrate only modest improvements in outcomes in effectiveness trials [3], a gap that, it is increasingly suspected, may be partially explained by a phenotype of intestinal abnormalities known as environmental enteric dysfunction (EED) [4], which is gaining recognition as a neglected disease [5]. According to the EED hypothesis, concurrent exposures to multiple enteric pathogens in already undernourished children cause cumulative damage to their guts’ surface, increasing its permeability to microbes and large molecules, causing systemic inflammation and impairing uptake and utilization of nutrients [6–8], which in turn leads to sub-optimal growth [9].

Studying the impact of EED is challenging. Gold standard diagnostic tests for other enteropathies, such as celiac and Crohn’s disease, include endoscopy and gut biopsy, invasive and demanding procedures that cannot feasibly be deployed in resource-constrained settings or to assess disease burden at the population level [10]. For this reason, there is considerable interest in identifying and validating biomarkers of EED that can be used as surrogate endpoints in population-based studies and for evaluating nutrition and hygiene interventions [11]. The most widely adopted biomarkers of EED use saccharide-based permeability assays like the lactulose/mannitol test [12]. However, such tests, while non-invasive, have well-documented limitations to their use in EED-endemic populations, taking hours to administer, requiring samples to be shipped to well-equipped facilities which makes them cumbersome, expensive and impractical for screening and randomization for intervention trials [13]. Several fecal biomarkers, such as alpha-1-antitrypsin, myeloperoxidase and neopterin, have been shown to have complex associations with growth outcomes [9,11], while certain plasma biomarkers show correlations with suboptimal growth, including the amino acid tryptophan and its ratio to its derivative, kynurenine [14].

Recently developed methods allow for quantifying large panels of soluble analytes in blood that relate to inflammation or immune status [15], however there is a lack of consensus about how to select the most important markers from among these panels and quantify their association and explanatory power with respect to specific disease outcomes relevant to EED (growth, cognitive function, immune activation, intestinal permeability, nutrient bioavailability, and hormones that alter growth and metabolism) [11,16]. Machine learning approaches have been used in biomarker analyses to identify best subsets of predictors from among large databases of candidate markers [16–18]. More specifically, penalized regression methods estimate coefficient values for modeled variables, while applying different penalties to those that overly increase model complexity relative to improving goodness of fit, assigning such variables a coefficient value of (“shrunk” to) zero. Those variables that are assigned non-zero coefficients can be interpreted as belonging to the subset that best predicts the outcome. Although these methods do not themselves report standard errors or adjust for within-cluster correlation in longitudinal data, the selected subsets can be included in more traditional multivariate regression models once identified and the effect size described by conventional methods.

The objective of this study was to identify clinically relevant biomarkers of the precursors of EED that can inform intervention early in the disease process. To this end, penalized regression approaches for variable subset selection were applied to a large panel of candidate biomarkers measured in a cohort of Peruvian infants to identify the optimal subset that are most predictive of nutritional status (length-for-age Z-score–LAZ-score) over a two-month lag.

## Methods

### Ethical approval and consent to participate

Ethical approval for MAL-ED was given by the Johns Hopkins Institutional Review Board as well as the Ethics Committee of Asociacion Benefica PRISMA, and the Regional Health Department of Loreto. Written informed consent was obtained from the caregiver of every participating child.

### Study population

A cohort of 303 infants was enrolled between December 2009 and February 2012 from Santa Clara de Nanay, a peri-urban community located 15 km from the city of Iquitos, Peru, a study setting that has been described in detail elsewhere [19]. Singleton births from a selected geographic area were enrolled within 17 days of birth provided they had no recognized congenital defects and weighed >2.4 kg at birth [20] and were followed up until 5 years of age. Daily data relating to infant feeding were ascertained by caregiver report from twice-weekly household visits from age 0 to 24 months, while anthropometric data and biological samples were collected during monthly assessments according to pre-established schedules [20].

### Outcome variable

The outcome of interest in this analysis was the subjects’ LAZ-score, a widely used measure of nutritional status and attained statural growth [21–23] that were calculated using WHO Anthro version 3.2.2. Anthropometric assessments were carried out at monthly intervals counted from the subjects’ birth dates from enrolment until 5 years of age. During these assessments, infants’ lengths were measured on marked platforms with a sliding footboard employing quality control measures that have been described elsewhere [23]. The LAZ-score was treated as a continuous variable. Its distribution in this study population has also been described elsewhere [11].

### Exposure variables

The primary exposure variables were 180 time-varying candidate fecal, urinary and plasma biomarkers of EED compiled from the following panels (biomarker names, abbreviations and units are listed in the supporting information):

1. Three overlapping panels of in-house quantitative, multiplexed immunoassays of cytokines, chemokines, hormones and other regulators of metabolism and growth [24], each run at the Myriad RBM laboratories (Austin, TX) on a separate subset of blood samples from the cohort including:
  - 86 analytes from 20 samples taken at age 7 months from a sub-sample of subjects and run in June 2013. These 20 subjects (10 cases and 10 controls) were selected for more expansive testing to examine extremes in growth in this setting over the target period between 6–15 months when exclusive breastfeeding is no longer the optimal feeding practice. Cases (positive deviants) were those subjects who grew by >0.77 LAZ, while controls (negative deviants) were selected from those subjects who experienced a change in LAZ of <0.25 over the 8-month follow-up period. This sub-sample was selected for a separate study to compare extremes in growth in this setting over the same period. At the age of 7 months, both cases and controls had equal LAZ.
  - 49 analytes from 178 samples mostly taken at the target age of 24 months (though with a small number taken at 7, 15, 25 and 26 months) run in May 2014.
  - 59 analytes from 443 samples taken at the target ages of 7 and 15 months (though with a small number taken at 8–9 or 16–18 months) run in January 2015.
2. 9 chemokine and 9 proinflammatory assays run on 596 of the same blood samples as 1 a-c at a laboratory at Johns Hopkins University (Baltimore, MD) in 2013–2014.
3. The amino acids citrulline and tryptophan and the latter’s metabolite kynurenine (umol/L) quantified in 640 of the same blood samples by liquid chromatography-mass spectrometry (LCMS) in the Oregon Analytics laboratory in 2015 [14].
4. 51 other biogenic amines quantified in 464 of the same blood samples by LCMS at a laboratory in Imperial College London in 2017 [25].
5. Several plasma analytes measured in the same blood samples as part of the MAL-ED protocol, including Alpha-1-acid glycoprotein (AGP—mg/dl, measured by radioimmune diffusion assay in 618 samples), Insulin-like growth factor (IGF) 1 and IFG-binding protein 3 (IGFBP-3—measured by enzyme-linked immunosorbent assay (ELISA) in 566 and 597 samples respectively) and hemoglobin (g/dL, measured by Hemocue).
6. Three fecal biomarkers—alpha-1-antitrypsin (AAT–mg/g), myeloperoxidase (MPO–ng/mL) and neopterin (NEO–nmol/L)—measured by ELISA tests of stool samples collected from the infants at monthly intervals [26].
7. 5 urinary biomarkers calculated from lactulose to mannitol recovery tests of intestinal permeability performed on urine samples collected at 3, 6, 9 and 15 months of age.

**Table 1** shows the number of biological samples and analytes available in each panel by age of the subjects. In addition, the following variables were included as potential confounders: infants’ sex, birthweight, breastfeeding status on the previous day (a time-varying categorical variable with four categories—“exclusively breastfed”, “partially breastfed”, “predominantly breastfed” and “not breastfed”), age in whole months (modeled using linear and quadratic terms) and mother’s height at the time of birth.

**Table 1:** Number of biological samples and analytes available in each panel included in the study by age at which they were taken.

		Panel number											
		1			2	3	4	5				6	7
		a.	b.	c.				AGP	IGF-1	IGFBP-3	Hb		
**Age in months**	**6**	0	0	0	0	0	0	0	0	0	0	174	267
	**7**	20	5	211	210	226	148	236	175	202	340	262	2
	**8**	0	0	2	2	7	2	2	2	1	7	264	1
	**9**	0	0	2	2	3	2	2	1	2	6	175	247
	**10**	0	0	0	0	0	0	0	0	0	0	257	3
	**11**	0	0	0	0	0	0	0	0	0	0	248	0
	**12**	0	0	0	0	0	0	0	0	0	0	253	0
	**13**	0	0	0	0	0	0	0	0	0	0	209	0
	**14**	0	0	0	0	0	0	0	0	0	0	203	0
	**15**	0	6	211	189	209	156	200	179	183	355	177	226
	**16**	0	0	13	10	11	12	12	12	11	14	213	1
	**17**	0	0	3	2	3	2	2	2	3	5	214	2
	**18**	0	0	1	1	1	1	1	1	1	2	226	0
	**19**	0	0	0	0	0	0	0	0	0	0	221	0
	**20**	0	0	0	0	0	0	0	0	0	0	218	0
	**21**	0	0	0	0	0	0	0	0	0	0	213	0
	**22**	0	0	0	0	0	0	0	0	0	0	206	0
	**23**	0	0	0	0	0	0	0	0	0	0	197	0
	**24**	0	167	0	167	167	129	154	181	182	304	182	180
	**25**	0	9	0	9	9	8	8	9	8	13	52	4
	**26**	0	4	0	4	4	4	1	4	4	4	43	1
**Total**		**20**	**191**	**443**	**596**	**640**	**464**	**618**	**566**	**597**	**1,050**	**4,207**	**934**
**Analytes**		**86**	**49**	**59**	**18**	**3**	**53**	**1**	**1**	**1**	**1**	**3**	**5**

### Statistical analysis

The fecal and urinary samples were matched to the plasma biomarker values that were closest in age and those that were not matched to any blood sample were excluded from the analysis. Exposure values were lagged by two months so that the analysis assessed the association between the subjects’ LAZ-score at month of age *j* and the exposures measured at age *j*-2 months. A 2-month lag was chosen because it is a length of time at which the impacts on a child’s growth of interventions such as steroids [27], chemotherapy [28] or treatment for severe acute malnutrition [29] become manifest, and therefore offers a feasible time window for clinical intervention and in which to reproducibly detect meaningful changes in ponderal growth associated with important physiologic determinants. Two months has also been demonstrated to be optimal for predicting future growth trajectory using fecal biomarkers [11]. All biomarkers were log-transformed with base 2. Because numerous biomarkers were either only available for samples collected at 24 months of age, or only for those collected around 7 and 15 months of age, the following analyses were performed on two subsets of the full biomarker database:

1. 7–15-month database–This excluded the samples in panel 1.b and any samples from panels 2–7 that were taken at ≥24 months of age, resulting in 461 observations and 110 biomarkers.
2. 7–24-month database–This included the 24-month samples but only for those biomarkers that were included in both panels 1.b and 1.c as well as panels 1. a and 2–7, resulting in 639 observations and 80 biomarkers.

#### Missing data

Non-detectable biomarker values, for which the analyte concentration was below the lower limit of quantification (LLOQ), were substituted with LLOQ /√2 [30]. No standard equivalent approaches exist for substituting values that are above the upper limit of quantification (ULOQ), however this only affected a small number of values for biomarkers in panel 4 which were treated as missing values. Almost all biomarkers and subjects had some number of missing values. Biomarkers for which more than 40% of the original values were missing were excluded from the imputation and further analysis, as were variables with fewer than 25 unique values within the detectable range. Observations that had missing values for more than 40% of the remaining biomarkers were excluded from the analysis. A small number of missing length measurements (n = 19, 3.0% of total) were linearly interpolated and extrapolated based on the actual or target date of assessment before calculating LAZ-scores. For time-fixed baseline variables (birth weight and maternal height), the small number of missing values were substituted with the sample mean of that variable. All other missing values of the biomarker exposures were imputed using multivariate normal regression (MVN) with an iterative Monte Carlo method to accommodate the arbitrary missing-value patterns of the continuous variables [31]. Missing values–of which there were 5,279 (11.1%) in the 7-15-month database and 4,508 (10.6%) in the 7–24-month database—were substituted with the average of the imputed values from 10 MVN imputations. The kynurenine/tryptophan (K/T ratio) and lactulose/mannitol ratios were excluded from imputation and recalculated after from their component biomarkers.

#### Variable selection

The retained biomarkers were included in penalized linear regression models with three different shrinkage methods that have been used in other studies of EED biomarkers—Adaptive LASSO (Least Absolute Shrinkage and Selection Operator), Minimax Concave Penalty (MCP) and Smoothly Clipped Absolute Deviation (SCAD) penalties [16,17]–with values for the tuning parameter *λ* determined through 10-fold cross validation. For each model, the variables assigned non-zero coefficients were treated as the optimal, best-predicting subset and the subset for the method that yielded the lowest cross-validation error (calculated from the mean-squared error or deviation from the fitted mean) was retained in a final multi-variable model.

#### Effect modeling

Regression models were fitted with robust variance estimation to allow for intra-subject correlation first for each of the candidate biomarkers separately (adjusting for the a priori-selected non-biomarker covariates) in order to report their independent effects and statistical significance and then for a multi-variable model that included all biomarkers selected for the best-fitting subset, to estimate the adjusted effect of each in the presence of the others and their combined effect on LAZ-score. To account for the false discovery rate (FDR) due to the large number of comparisons, *p*-values from the separately modeled biomarkers were compared visually in scatterplots with their corresponding *q*-values (a measure of significance in terms of the FDR [32] calculated using the method proposed by Simes [33]) and with a Bonferroni corrected *α* value calculated from the number of comparisons. The effect measures from the single-biomarker and adjusted subset models were visualized using forest plots. For the biomarkers included in the final, multi-variable models, the coefficient estimates were reported along with the difference in a child’s height predicted by the model between subjects at the 25^th^ and the 75^th^ percentile of each included biomarker’s distribution at the age of the final included sample (15 or 24 months, depending on which database was used) and holding all other included biomarkers at their mean values and based on the standard deviation in height at that age reported in the WHO child growth standards [21]. The *R*^2^ statistic for the final subset model was reported along with the partial *R*^2^ for all included biomarker terms as an estimate of the proportion of the total variability in the outcome that was explained by the selected biomarker subset. Results from the final models were compared with those obtained from adjusting for LAZ-score measured contemporaneously with the biomarker (in place of the other covariates), in order to compare the prognostic potential of the final biomarker subset in predicting future growth relative to a natural and existing alternative, namely attained LAZ-score. The potential for non-linear relationships between biomarkers in the final subsets and LAZ-score was assessed by generating nonparametric smooth plots and by applying a multivariate spline model-selection algorithm to the final models. Finally, as a validation exercise, associations between each of three of the most important biomarkers (expressed per standard deviation [SD]) and changes in LAZ over increasing lag-lengths of 1–10 months adjusting for contemporaneous LAZ were plotted to assess the performance using an existing method that has previously been used for tryptophan and citrulline and compared to a comparator biomarker of a known endocrinologic agent–Insulin-like growth factor 1 (IGF-1), replicating the methodology of Kosek and colleagues [14]. Analyses were carried out using Stata 15.1 [34] and R 3.6.1.

## Results

Summary statistics of the distributions of the 180 candidate biomarkers and whether they met the criteria for inclusion in further analysis are presented in **S1 Table** in the supporting information. A participant flowchart is provided as **S1 Fig** (supporting information). Before applying exclusion criteria, 639 observations were available for 258 of the 303 enrolled subjects for whom blood samples were available relating to 180 biomarkers. 23 of the biomarkers were only available in the case control panel (panel 1a.) and so were excluded from further analysis for only having 20 available observations. A further 47 biomarkers were excluded from the 7-15-month database either because more than 40% of their values were missing, fewer than 25 were within the detectable range, they were only available at 24 months of age (panel 1c.) or some combination of these. 77 biomarkers were excluded from the 7-24-month database due to missingness, detectability, or because they did not have values available at 24 months. Overall, 110 biomarkers were retained for analysis in the 7-15-month database, and 80 in the 7-24-month.

**Table 2** shows the number of biomarkers selected (assigned non-zero coefficients) and the cross-validation error and R^2^ values for the three penalized regression models fitted on each of the two biomarker databases. MCP selected the smallest subset of biomarkers when fitted to the 7-15-month, but not the 7-24-month database, for which adaptive LASSO selected the smallest. For both databases, the SCAD penalty resulted in the largest subset, the highest cross-validated R^2^ and the lowest cross-validation error (jointly with MCP in the 7–24 month model) and was chosen as the subset for subsequent analyses.

**Table 2:** Number of biomarkers selected (assigned non-zero coefficients) and cross-validation error for three penalized regression models fitted on two biomarker databases.

	7–15 months			7–24 months		
	Adaptive LASSO	MCP	SCAD	Adaptive LASSO	MCP	SCAD
**Biomarkers selected**	17	8	23	5	22	25
**Cross-validation error**	0.84	0.84	0.82	0.85	0.78	0.78
**Cross-validated R-squared**	0.06	0.05	0.08	0.10	0.10	0.10

**Fig 1:**
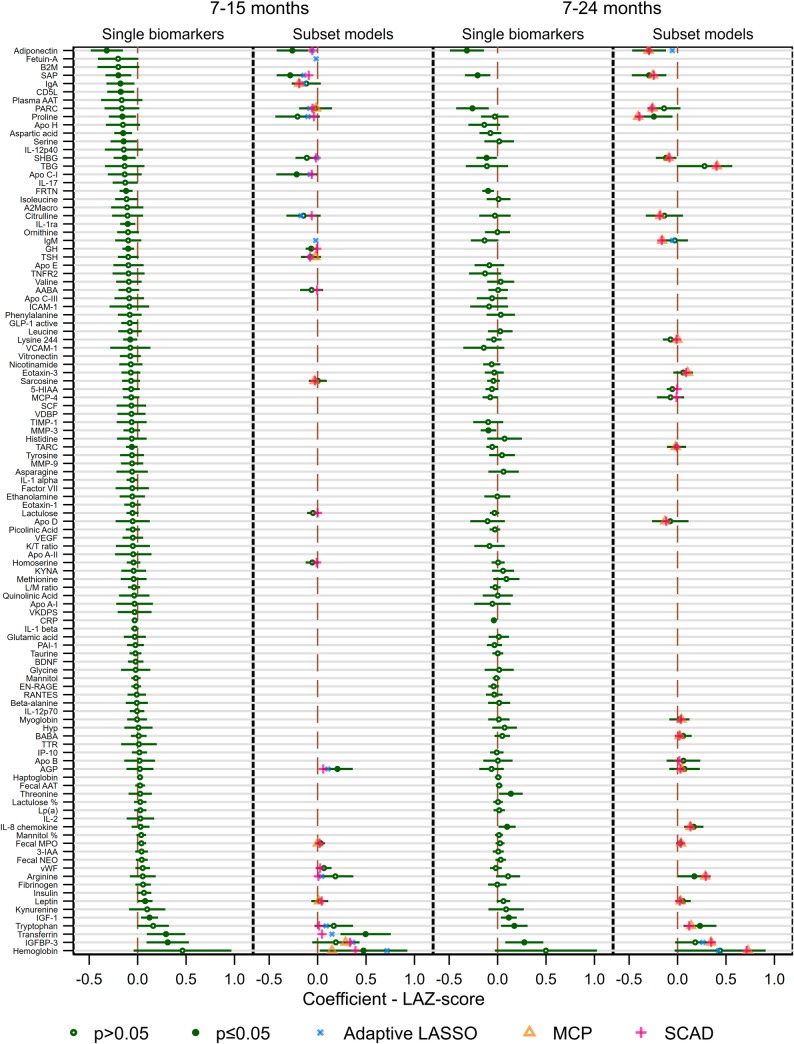
Forest plots of coefficient estimates and 95% confidence intervals from linear regression models of single biomarkers, for subsets of multiple biomarkers selected by the three penalized regression methods and from a final multi-variable linear regression model of the subset with the lowest cross-validation error adjusting for covariates.

**Fig 1** shows the coefficient estimates from the linear regression models of single biomarkers, for the subsets of multiple biomarkers selected by the three penalized regression methods and from fitting a final multi-variable linear regression model to the SCAD-selected subset–the penalty with the lowest cross-validation error—adjusting for covariates. For 82 of the 110 biomarkers in the 7-15-month database, the single biomarker model predicted a negative association with the outcome, compared with 28 for which a positive association was predicted. In 17 of these models, the estimate was statistically significant at the uncorrected *α* = 0.05 level. 42 of the 80 biomarkers in the 7-24-month database had a negative association with the outcome in the single biomarker models, 38 had a positive association, and 12 had statistically significant estimates. No fecal or urinary biomarkers were included in the final subsets selected by adaptive LASSO, although in both the 7–15 and 7-24-month models, the SCAD and MCP penalties assigned small, non-zero coefficients to fecal MPO and SCAD also selected urinary lactulose.

In both databases, just 5 biomarkers were selected by all three penalties. In the 7-15-month models, these were hemoglobin, Immunoglobulin A (IgA), Insulin-like growth factor-binding protein 3 (IGFBP-3), Pulmonary and Activation-Regulated Chemokine (PARC) and Thyroid-Stimulating Hormone (TSH), while in the 7-24-month models these included adiponectin and IgM instead of IgA and TSH. In both databases, all biomarkers selected by MCP were also selected by SCAD and in the 7-24-month database all biomarkers selected by adaptive LASSO were also selected by the other two penalties. SCAD selected 5 biomarkers in the 7-15-months and 3 in the 7-24-month database that were not included in either of the other two subsets, however only one of these–growth hormone (GH)–was significant in the final model.

**Fig 2:**
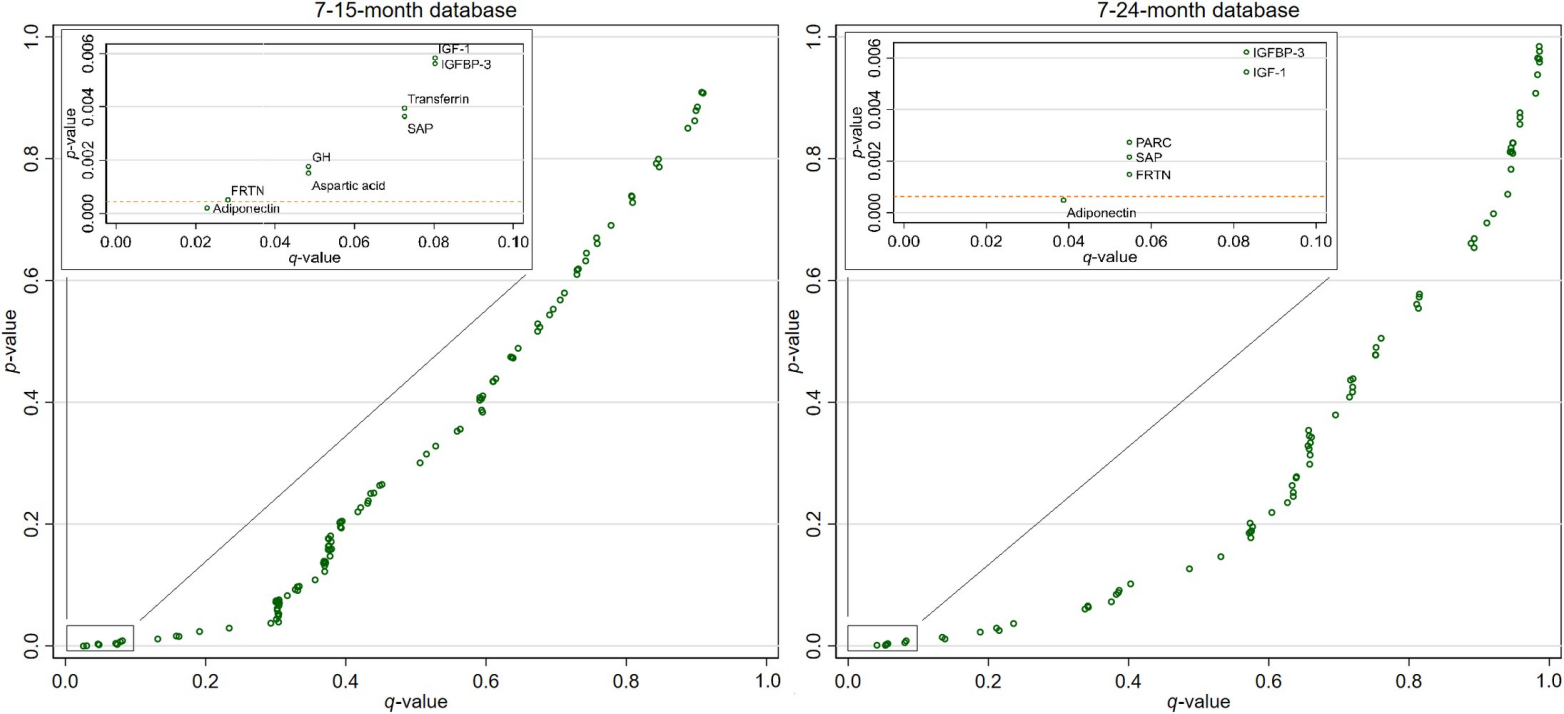
Scatterplot comparing the *p*-values from the separately modeled biomarkers to their corresponding *q*-values calculated using the method proposed by Simes’ method [33] and to the Bonferroni corrected *α* values (represented by the dashed lines). Biomarkers for which *q*<0.1 are labeled.

**Fig 2** plots the *p*-values from the separately modeled biomarkers against their corresponding *q*-values with lines representing the Bonferroni corrected *α* values to assess their significance after adjusting for the FDR. For both databases, only adiponectin retained statistical significant at the Bonferroni corrected *α* levels, while a small number of other biomarkers–Ferritin (FRTN), IGF-1, IGFBP-3 and Serum Amyloid P-Component (SAP) in both databases, GH and aspartic acid for the 7-15-month data and PARC for the 7-24-month–had *q*-values below the less conservative threshold of *q*<0.1.

**Table 3** presents the coefficient estimates from the final 7-15-month and 7-24-month linear regression models for a 1 log_2_ increase of each of the biomarkers selected by SCAD along with the difference in child’s height predicted for children aged 17 and 26 months respectively at the 25^th^ and 75^th^ percentile of the biomarker distribution (holding all other included biomarkers at their sample mean). The 36 selected biomarkers include numerous amino acids, chemokines, hormones, glycoproteins and proteins along with two antibodies, three apolipoproteins, the enzyme myeloperoxidase, the sugar lactulose and 5-OH-Indole-3-acetic Acid (5-HIAA), the metabolite of serotonin. Thirteen biomarkers were included in both final models, while 11 were only included in the 7-15-month model and 12 only in the 7-24-month model.

**Table 3:** Coefficient estimates (with 95% confidence intervals) from linear regression models for biomarkers selected by SCAD along with the predicted difference in child’s height 2 months after the last sample for children at the 25^th^ and 75^th^ percentile of the biomarker distribution.

Biomarker	7 & 15 months		7, 15 & 24 months	
	Coefficient—LAZ score	Predicted height difference (cm) at 17 months	Coefficient—LAZ score	Predicted height difference (cm) at 26 months
**5-OH-Indole-3-acetic Acid (5-HIAA)**	-	-	-0.05 (-0.11, 0.00)	-0.16
**Alpha-2-Macroglobulin (A2Macro)**	-0.07 (-0.25, 0.11)	-0.10	-	-
**Alpha-amino-n-butyric acid (AABA)**	-0.06 (-0.18, 0.06)	-0.15	-	-
**Adiponectin**	-0.26 (-0.42, -0.10)	-0.40	-0.29 (-0.47, -0.12)	-0.54
**alpha-1-acid glycoprotein (AGP)**	0.20 (0.05, 0.36)	0.33	0.07 (-0.09, 0.23)	0.14
**Apolipoprotein B (Apo B)**	-	-	0.06 (-0.11, 0.23)	0.13
**Apolipoprotein C-I (Apo C-I)**	-0.22 (-0.43, -0.01)	-0.30	-	-
**Apolipoprotein D (Apo D)**	-	-	-0.08 (-0.26, 0.11)	-0.12
**Arginine**	0.18 (-0.00, 0.37)	0.39	0.17 (0.00, 0.34)	0.46
**Beta-amino-iso-butyric acid (BABA)**	-	-	0.06 (-0.03, 0.15)	0.38
**Citrulline**	-0.15 (-0.32, 0.03)	-0.22	-0.14 (-0.33, 0.06)	-0.24
**Eotaxin-3**	-	-	0.06 (-0.04, 0.16)	0.11
**Fecal Myeloperoxidase (MPO)**	0.03 (-0.01, 0.08)	0.19	0.03 (-0.02, 0.07)	0.20
**Growth Hormone (GH)**	-0.07 (-0.12, -0.01)	-0.28	-	-
**Hemoglobin**	0.47 (0.02, 0.93)	0.30	0.44 (-0.03, 0.91)	0.31
**Homoserine**	-0.05 (-0.12, 0.01)	-0.16	-	-
**Immunoglobulin A (IgA)**	-0.12 (-0.27, 0.03)	-0.23	-	-
**Immunoglobulin M (IgM)**	-	-	-0.03 (-0.16, 0.11)	-0.07
**Insulin-like growth factor-binding protein 3 (IGFBP-3)**	0.19 (-0.06, 0.43)	0.25	0.18 (-0.02, 0.39)	0.28
**Interleukin-8 (IL-8) chemokine**	-	-	0.17 (0.07, 0.27)	0.63
**Lactulose**	-0.05 (-0.11, 0.01)	-0.24	-	-
**Leptin**	0.02 (-0.07, 0.11)	0.09	0.06 (-0.02, 0.14)	0.27
**Lysine 244**	-	-	-0.07 (-0.15, 0.01)	-0.21
**Monocyte Chemotactic Protein 4 (MCP-4)**	-	-	-0.07 (-0.21, 0.07)	-0.25
**Myoglobin**	-	-	0.02 (-0.08, 0.12)	0.06
**Pulmonary and Activation-Regulated Chemokine (PARC)**	-0.02 (-0.19, 0.15)	-0.04	-0.14 (-0.31, 0.03)	-0.28
**Proline**	-0.21 (-0.44, 0.02)	-0.42	-0.24 (-0.44, -0.05)	-0.50
**Serum Amyloid P-Component (SAP)**	-0.28 (-0.42, -0.14)	-0.65	-0.29 (-0.47, -0.12)	-0.79
**Sarcosine**	0.00 (-0.09, 0.09)	0.01	-	-
**Sex Hormone-Binding Globulin (SHBG)**	-0.11 (-0.23, 0.01)	-0.33	-0.12 (-0.22, -0.01)	-0.42
**Thymus and activation regulated chemokine (TARC)**	-	-	-0.01 (-0.11, 0.09)	-0.05
**Thyroxine-Binding Globulin (TBG)**	-	-	0.28 (-0.01, 0.56)	0.36
**Transferrin**	0.50 (0.24, 0.75)	0.66	-	-
**Tryptophan**	0.17 (-0.03, 0.37)	0.29	0.23 (0.06, 0.40)	0.44
**Thyroid-Stimulating Hormone (TSH)**	-0.07 (-0.17, 0.03)	-0.16	-	-
**von Willebrand Factor (vWF)**	0.07 (-0.01, 0.14)	0.23	-	-

The iron-transporting glycoprotein transferrin had the largest effect size in the 7-15-month model both in terms of its estimated coefficient–a highly statistically significant 0.50 (0.24, 0.75) increase in the predicted LAZ-score–and the height difference predicted–a 17-month-old child at the 75^th^ percentile of plasma transferrin concentration being two thirds of a centimeter taller than one at the 25^th^. Hemoglobin had the second largest absolute coefficient value in the 7-15-month model–a slightly significant 0.47 (0.02, 0.93)–but the second largest difference in height was predicted by SAP—a child at the 3rd quartile of its distribution predicted to be 0.65 cm shorter than one at the 1st quartile–which also had a highly statistically significant coefficient estimate. Other biomarkers for which the 7-15-month model predicted large and statistically significant negative effects include the hormones adiponectin and GH–predicting respectively around a -0.4cm and a -0.28cm height difference–and apolipoprotein (Apo) C-I—-0.3cm–while AGP had a slightly statistically significant positive effect.

Several biomarkers that had large effect sizes in the 7-15-months model–transferrin, Apo C-I and GH- were not included in the 7-24-month database, due to no values being available at 24 months of age. Instead, in that model, while hemoglobin again had the largest coefficient estimate–a non-significant 0.44 (-0.03, 0.91)–SAP predicted the largest difference in height between the extremes of the interquartile range of the analyte’s distribution at 24 months– 26-month-old children with high SAP concentration at 24-months a predicted 0.79cm shorter than their low SAP counterparts–the next largest being the chemokines Interleukin-8 (IL-8)– 0.63cm taller–and adiponectin– 0.54cm shorter–the latter having a highly statistically significant effect estimate. Proline, arginine, tryptophan and SHBG also all had slightly statistically significant coefficient estimates and predicted among the largest height differences.

The final 7-15-month model explained 43.0% of the variance in the LAZ-score according to the *R*^2^ statistics, with 23.0% of the variance explained solely by the selected subset of biomarkers (the partial *R*^2^ statistic excluding the non-biomarker covariates). The equivalent proportions for the final 7-24-month model were 39.6% and 17.7% respectively. **S2 Table** in the supporting information show the equivalent results when the non-biomarker covariates were replaced in the final models with contemporaneous LAZ-score to adjust for attained growth. In the presence of this variable, many of the effect size estimates decreased in magnitude and statistical significance considerably including transferrin and GH in the 7-15-month model, tryptophan and SHBG in the 7-24-month model and adiponectin and SAP in both models. Several biomarkers did increase in statistical significance upon adjustment for attained growth however, including Alpha-2-Macroglobulin (A2Macro), fecal MPO, tryptophan and TSH in the 7-15-month and proline and hemoglobin in the 7-24-month models. Adjustment for baseline LAZ-score also greatly increased the proportion of the variability explained by the models—R^2^ statistics of 83.7% and 84.3% for the 7-15-month and 7-24-month models respectively–but decreased the proportion explained by the biomarker subsets– 9.6% and 7.3% respectively—demonstrating that growth already attained has far more explanatory power for modeling short-term future growth than any combination of biomarkers.

Numerous biomarkers, including Eotaxin-3, citrulline, myoglobin, lactulose, and SHBG, exhibited evidence of having non-linear relationships with the outcome when visualized in polynomial smooth plots (S2–S6 Figs respectively in the supporting information). When a multivariate spline model-selection algorithm was run on each of the two final biomarker subsets, none of the biomarkers improved the model when represented by multiple cubic splines relative to linear terms with the exceptions of proline in the 7-15-month model (4 degrees of freedom) and Thymus and activation regulated chemokine (TARC) and Monocyte Chemotactic Protein 4 (MCP-4) (2 degrees of freedom each) in the 7-24-month model (results not reported).

**Fig 3:**
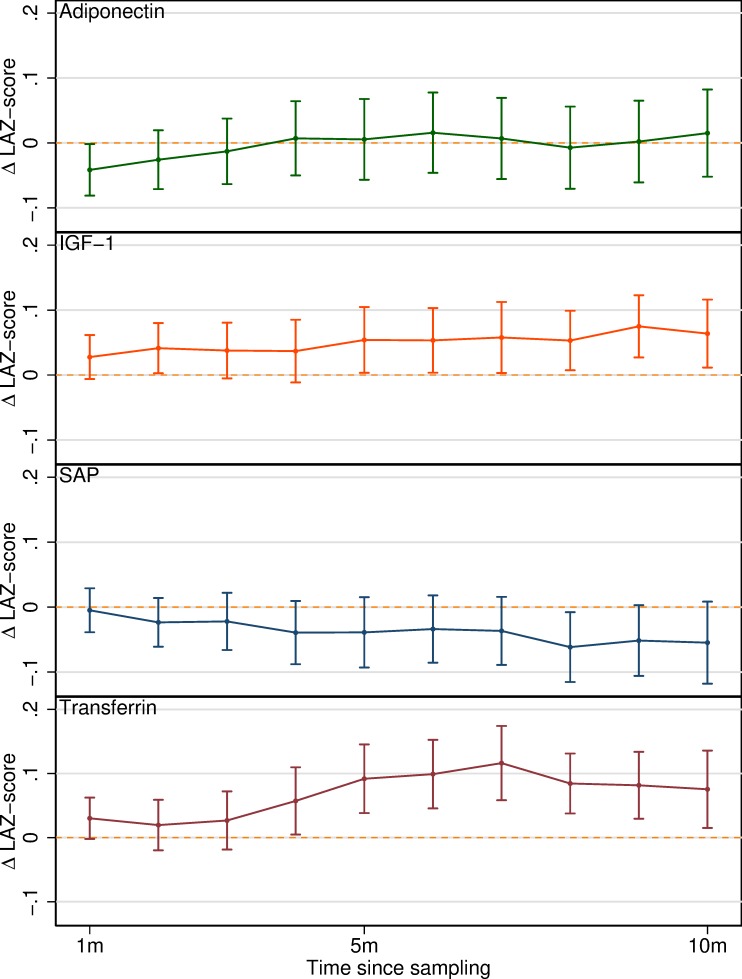
Associations between concentrations of four plasma biomarkers (per standard deviation of their log_2_ transformed values) and differences in LAZ-score (ΔLAZ) over time windows of increasing length, from models adjusting for baseline LAZ, age, and sex.

**Fig 3** shows the results of the validation exercise in which a previously published methodology was replicated using three biomarkers from the final subset identified here along with IGF-1 as a comparator. This analysis treated the difference in LAZ-score (ΔLAZ) over time-windows of increasing length as the outcome, standard deviations of the biomarkers as exposures and adjusted for baseline LAZ, as well as the other covariates. Adiponectin, which had previously exhibited a large and highly statistically significant association with nutritional status showed no obvious trend after adjustment for attained growth, while IGF-1 and, most markedly, transferrin showed large and statistically significant associations with changes in LAZ-score over longer time windows of 5–10 months.

## Discussion

Analytical techniques such as multiplex immunoassays and mass spectrometry are increasingly being used in human studies to enable the quantification of ever more diverse and extensive panels of analytes in biological samples, many of which have biological functions that have yet to be fully characterized. At the same time, advanced statistical learning methods have emerged that can be used to identify patterns in large datasets. This study brings together these two developments and applies them to an issue that has received growing attention in recent years but has yet to be fully resolved–identifying prognostic biomarkers of EED that can predict future linear growth over time windows relevant to clinical intervention. In a birth cohort recruited from a low-resource setting in Peru, this study reports the distributions of 180 candidate biomarkers in fecal, urinary and plasma samples, of which 110 met the criteria for inclusion in variable-subsetting penalized regression models–the largest number of markers ever considered in a study of this nature.

The final subsets selected by SCAD penalty included numerous biomarkers that previous studies have implicated as potential predictors of linear growth and markers of gut function. The essential amino acid tryptophan has previously shown promise as a prognostic indicator of EED due to its role in normal infant growth and its hypothesized correlation with indoleamine 2,3-dioxygenase 1 (IDO1) activity in states of chronic low-grade endotoxin exposure [14]. However, while tryptophan was selected by the majority of the penalized regression models, and its association with LAZ-score was statistically significant in the 7-24-month final models it was not among the biomarkers most predictive of differences in height. A positive association between plasma tryptophan concentration and a 6-month change in LAZ-score has already been reported in this cohort and a similar one in Tanzania and separately in one in Northeast Brazil with effect sizes comparable to that of the final model here [14,35]. Immunoglobulin A (IgA), which was retained in the final 7-15-month model, had a small, non-significant, negative effect size consistent with that observed for IgA anti-LPS antibody also in the Brazil cohort [35].

For some other biomarkers in the subsets, evidence in previous literature on EED is more scant though known mechanisms nonetheless exist through which they might plausibly track nutritional status. Most obvious of these is hemoglobin, long the gold standard marker of severe anemia and therefore of its attendant delaying effects on growth and development [36]. Analysis of data from the 8-site study to which the cohort described here contributed found an association (though weaker and less significant than those found here) between hemoglobin and LAZ-score at age 5 years [37], while other studies of EED have adjusted for hemoglobin as a potential confounder [38,39]. Low levels of plasma transferrin are found during protein-energy malnutrition [40]. Adiponectin is an appetite-regulating hormone that promotes satiety and therefore may inhibit food intake, which may explain its negative association with growth [41,42]. While elevated levels of circulating adiponectin have a known negative association with obesity [43], its role in child growth is unclear, and among twins this adipokine had a positive association with birthweight-adjusted LAZ-score (counter to the negative one reported here) [42]. Leptin and the serum leptin-adiponectin ratio were found to be associated with stunting in Bangladeshi children and increased in this group following food supplementation [38]. The positive association between serum arginine concentrations and nutritional status is consistent with findings from Malawi, though the same study failed to find a significant association with proline, which was one of the more predictive of the biomarkers in these results [44].

While the SCAD-selected subset was used for the final models due to its yielding the low cross-validated error and explaining a larger proportion of the variance, it is notable that this penalty did select several biomarkers that had small non-significant effect estimates and did not select several biomarkers, which had statistically significant single biomarker effect sizes and known associations with nutritional outcomes (such as IGF-1 and ferritin). Though SCAD has been used in numerous studies of EED biomarkers [16–18], these findings do suggest that this penalty lacks both sensitivity and specificity when applied to large panels.

For other biomarkers in the subsets, the functions or pathways through which they might impact growth are as yet unclear, which demonstrates the hypothesis-generating potential of this approach. SHBG is of interest in biomarker research for its association at low levels with type-II diabetes and metabolic syndrome but, although elevated SHBG is seen following weight loss, this glycoprotein has not previously been considered as a prognostic marker of growth faltering [45]. Though known for its association with amyloidosis, SAP is also involved in the humoral innate immune system’s response to infections and might plausibly lie on the pathway connecting enteric pathogen infection to growth deficits that is specific to the EED hypothesis [46–48]. TBG, responsible for binding the thyroid hormones thyroxine and triiodothyronine in the blood down, which downregulate the activity of hormones that stimulate metabolic rate and may influence the regulation of skeletal growth [49,50].

C-Reactive Protein (CRP), which multiple previous studies have found to be a promising biomarker [17,51], was not selected despite having a statistically significant, though small, negative effect in the single biomarker 7–24 months model. The fact that CRP is inversely related to Fetuin-A [52] and, like SAP, is a calcium-dependent ligand binding plasma protein [46] may mean that the presence of the latter protein in the final model fully accounted for any effect of CRP. The three fecal biomarkers and the urinary lactulose/mannitol ratio (along with the other four urinary markers) have shown clinical potential in previous studies [11,53] but in this analysis were not significant in any of the single-biomarker or final models. It may be the case that restricting the data to assessments at just 2–3 time points meant that the analysis was underpowered to detect the true but relatively small effects of these substances [11]. Citrulline, which has shown promise in previous studies [35], was not significant in either single biomarker model, and was selected but not significant in the final models.

Although ferritin, the body’s stored form of iron, has been implicated previously [17,51] and was significant in the single biomarker model, it was not selected here for either final model. This may be because its association with growth is mediated by the stronger and more statistically significant effect of the related glycoprotein transferrin [54]. Some biomarkers that have been implicated in other studies–such as soluble CD14 [16,17], endotoxin core antibodies (EndoCAB) [12], zonulin, intestinal fatty acid binding protein [35], retinol binding protein and calprotectin [17]–were not included in any of the panels. Others were excluded from the analysis due to having too few unique observations, notably almost all the interleukins, which were only tested for in the case-control panel, a limitation of this study.

Several other limitations warrant highlighting. Most associations that were apparently statistically significant in the single biomarker models appeared much less so after accounting for the FDR–indeed, only adiponectin remained significant at the Bonferroni-corrected α level. Furthermore, the results of the adjusted subset models do not account for the variable selection in the first stage SCAD model, a post-selection inference problem that can lead to inflated type-1 errors and overly narrow confidence intervals [55]. However, the associations identified by this analysis should be assessed, not just by their statistical significance but by their biological plausibility and in light of the fact that the biomarkers selected for the subset and the relative strength of their associations with the outcome are broadly consistent with known biological pathways. Another limitation is the assumption both in the subset selection stage and in fitting the final models that any relationships between biomarkers and LAZ-score would be linear. Exploratory analysis revealed some evidence to challenge this, which may limit the accuracy of the predictions from the linear models, however further analysis using multivariate regression splines suggested that only a very small number of biomarkers were affected by this assumption. As consensus develops around a final set of important biomarkers of EED such non-linear effects will need to be more rigorously characterized.

Applying the penalized regression models to the database that included the observations at 24 months of age, did not improve the predictive capability of the model. Similarly, the final 7-24-month model explained a smaller proportion of the variance in the outcome than the 7-15-month model. However, for some biomarkers that were included in both models, the 7-24-month model tended to give larger and more statistically significant effect size estimates than the 7-15-month model (with the notable exception of hemoglobin). The reason for the difference in explanatory power may be because the 7-24-month database did not include transferrin (which was not tested at 24 months of age), the biomarker with the largest effect size in the 7-15-month final model.

Studies with more intensive sample collection and frequent follow-up are needed to explore random effects and short-term intra- and inter-subject variability of these biomarkers as well as those that were excluded from this analysis and to more precisely model their effects on growth [11]. The validity of these biomarkers as clinically relevant predictors of growth in new populations can be readily assessed given that ELISA kits for most of them are commercially available. This is important considering the high burden of stunting in under-resourced settings in low- and middle-income countries where these biomarkers can potentially be tested in regional laboratories, and the results used to inform care and programs aimed at controlling stunting.

The expanded testing of analytes chosen for their characterization as being important immune and metabolic regulators pertinent to child growth revealed several important findings. This selected subset of biomarkers explained 17.7–23.0% of the variance in LAZ score with measurements taken at 2 or 3 time points, compared to a single biomarker such as MPO which only accounted for 2.8% of the variance with monthly follow-up up to age 3 years in the same population [11]. Future studies should aim to characterize changes in LAZ scores when assessing the interaction between EED biomarkers and intestinal infections by specific pathogens. These plasma biomarkers represent a set of surrogate outcomes which can be measured at different time points, all of which are characteristic of a good biomarker of EED to circumvent the problems associated with the lactulose/mannitol test, the current gold standard test (such as the variable in its association with child growth, which, even when significant has an effect size that is much smaller than the selected panel described here) [56].

In summary, penalized regression modeling approaches–most notably SCAD—can be used to select subsets from large panels of candidate biomarkers of EED providing translational value in the form of further evidence for known markers and in generating hypotheses about new ones. Adiponectin, IL-8, proline, SAP and transferrin, among others, are promising plasma biomarkers of EED.

## Supporting information

S1 List Biomarkers quantified in each panel and their units. (PDF)

S1 Table Summary statistics of candidate biomarkers. (PDF)

S2 Table Coefficient estimates (with 95% confidence intervals) from linear regression models for biomarkers selected by SCAD along with the predicted difference in child’s height 2 months after the last sample for children at the 25^th^ and 75^th^ percentile of the biomarker distribution adjusted for contemporaneous LAZ-score. (PDF)

S3 Table STROBE checklist. (PDF)

S1 Fig Participant flowchart. (TIF)

S2 Fig Polynomial smooth plot of the relationship between plasma Eotaxin-3 concentration and lagged LAZ-scores. (TIF)

S3 Fig Polynomial smooth plot of the relationship between plasma citrulline concentration and lagged LAZ-scores. (TIF)

S4 Fig Polynomial smooth plot of the relationship between plasma myoglobin concentration and lagged LAZ-scores. (TIF)

S5 Fig Polynomial smooth plot of the relationship between urinary lactulose concentration and lagged LAZ-scores. (TIF)

S6 Fig Polynomial smooth plot of the relationship between plasma Sex Hormone-Binding Globulin concentration and lagged LAZ-scores. (TIF)

## References

[1] Black RE, Allen LH, Bhutta ZA, Caulfield LE, de Onis M, Ezzati M, et al Maternal and child undernutrition: global and regional exposures and health consequences. Lancet (London, England). 2008;371: 243–60. 10.1016/S0140-6736(07)61690-0 18207566

[2] Victora CG, de Onis M, Hallal PC, Blössner M, Shrimpton R. Worldwide timing of growth faltering: revisiting implications for interventions. Pediatrics. 2010;125: e473–80. 10.1542/peds.2009-1519 20156903

[3] Dewey KG, Adu-Afarwuah S. Systematic review of the efficacy and effectiveness of complementary feeding interventions in developing countries. Matern Child Nutr. 2008;4: 24–85. 10.1111/j.1740-8709.2007.00124.x 18289157 PMC6860813

[4] Harper KM, Mutasa M, Prendergast AJ, Humphrey J, Manges AR. Environmental enteric dysfunction pathways and child stunting: A systematic review. PLoS Negl Trop Dis. 2018;12 10.1371/journal.pntd.0006205 29351288 PMC5792022

[5] Arndt MB, Walson JL. Enteric infection and dysfunction—A new target for PLOS Neglected Tropical Diseases. Ryan ET, editor. PLoS Negl Trop Dis. 2018;12: e0006906 10.1371/journal.pntd.0006906 30592716 PMC6310236

[6] Kelly P, Menzies I, Crane R, Zulu I, Nickols C, Feakins R, et al Responses of small intestinal architecture and function over time to environmental factors in a tropical population. Am J Trop Med Hyg. 2004;70: 412–9. Available: http://www.ncbi.nlm.nih.gov/pubmed/15100456 15100456

[7] Korpe PS, Petri WA. Environmental enteropathy: critical implications of a poorly understood condition. Trends Mol Med. 2012;18: 328–36. 10.1016/j.molmed.2012.04.007 22633998 PMC3372657

[8] Kosek MN, Ahmed T, Bhutta Z, Caulfield L, Guerrant R, Houpt E, et al Causal Pathways from Enteropathogens to Environmental Enteropathy: Findings from the MAL-ED Birth Cohort Study. EBioMedicine. 2017;18: 109–117. 10.1016/j.ebiom.2017.02.024 28396264 PMC5405169

[9] Kosek M, Haque R, Lima A, Babji S, Shrestha S, Qureshi S, et al Fecal Markers of Intestinal Inflammation and Permeability Associated with the Subsequent Acquisition of Linear Growth Deficits in Infants. Am J Trop Med Hyg. 2013;88: 390–396. 10.4269/ajtmh.2012.12-0549 23185075 PMC3583335

[10] Keusch GT, Rosenberg IH, Denno DM, Duggan C, Guerrant RL, Lavery J V., et al Implications of Acquired Environmental Enteric Dysfunction for Growth and Stunting in Infants and Children Living in Low- and Middle-Income Countries. Food Nutr Bull. 2013;34: 357–364. 10.1177/156482651303400308 24167916 PMC4643688

[11] Colston JM, Peñataro Yori P, Colantuoni E, Moulton LH, Ambikapathi R, Lee G, et al A methodologic framework for modeling and assessing biomarkers of environmental enteropathy as predictors of growth in infants: an example from a Peruvian birth cohort. Am J Clin Nutr. 2017;106: 245–55. 10.3945/ajcn.116.151886 28592604

[12] Hoke MK, McCabe KA, Miller AA, McDade TW. Validation of endotoxin-core antibodies in dried blood spots as a measure of environmental enteropathy and intestinal permeability. Am J Hum Biol. 2018; e23120 10.1002/ajhb.23120 29532544

[13] Faubion WA, Camilleri M, Murray JA, Kelly P, Amadi B, Kosek MN, et al Improving the detection of environmental enteric dysfunction: a lactulose, rhamnose assay of intestinal permeability in children aged under 5 years exposed to poor sanitation and hygiene. BMJ Glob Heal. 2016;1: e000066 10.1136/bmjgh-2016-000066 28588929 PMC5321325

[14] Kosek MN, Mduma E, Kosek PS, Lee GO, Svensen E, Pan WKY, et al Plasma Tryptophan and the Kynurenine-Tryptophan Ratio are Associated with the Acquisition of Statural Growth Deficits and Oral Vaccine Underperformance in Populations with Environmental Enteropathy. Am J Trop Med Hyg. 2016;95: 928–937. 10.4269/ajtmh.16-0037 27503512 PMC5062803

[15] Breen EC, Reynolds SM, Cox C, Jacobson LP, Magpantay L, Mulder CB, et al Multisite comparison of high-sensitivity multiplex cytokine assays. Clin Vaccine Immunol. 2011;18: 1229–42. 10.1128/CVI.05032-11 21697338 PMC3147360

[16] Lu M, Zhou J, Naylor C, Kirkpatrick BD, Haque R, Petri WA, et al Application of penalized linear regression methods to the selection of environmental enteropathy biomarkers. Biomark Res. 2017;5: 9 10.1186/s40364-017-0089-4 28293424 PMC5345248

[17] Naylor C, Lu M, Haque R, Mondal D, Buonomo E, Nayak U, et al Environmental Enteropathy, Oral Vaccine Failure and Growth Faltering in Infants in Bangladesh. EBioMedicine. 2015;2: 1759–66. 10.1016/j.ebiom.2015.09.036 26870801 PMC4740306

[18] Moreau GB, Ramakrishnan G, Cook HL, Fox TE, Nayak U, Ma JZ, et al Childhood growth and neurocognition are associated with distinct sets of metabolites. EBioMedicine. 2019;44: 597–606. 10.1016/j.ebiom.2019.05.043 31133540 PMC6604877

[19] Yori PP, Lee G, Olortegui MP, Chavez CB, Flores JT, Vasquez AO, et al Santa Clara de Nanay: The MAL-ED Cohort in Peru. Clin Infect Dis. 2014;59: S310–S316. 10.1093/cid/ciu460 25305303

[20] MAL-ED Network Investigators The MAL-ED Network Investigators, MAL-ED Network Investigators. The MAL-ED study: a multinational and multidisciplinary approach to understand the relationship between enteric pathogens, malnutrition, gut physiology, physical growth, cognitive development, and immune responses in infants and children up to 2 years of. Clin Infect Dis. 2014;59 Suppl 4: S193–206. 10.1093/cid/ciu653 25305287

[21] WHO Multicentre Growth Reference Study Group. WHO Child Growth Standards: Length/height-for-age, weight-for-age, weight-for-length, weight-for-height and body mass index-for-age: Methods and development. Geneva: World Health Organization; 2006 Available: http://www.who.int/childgrowth/standards/technical_report/en/

[22] Kosek M, Guerrant RL, Kang G, Bhutta Z, Yori PP, Gratz J, et al Assessment of environmental enteropathy in the MAL-ED cohort study: theoretical and analytic framework. Clin Infect Dis. 2014;59 Suppl 4: S239–47. 10.1093/cid/ciu457 25305293 PMC4204611

[23] Richard SA, McCormick BJJ, Miller MA, Caulfield LE, Checkley W. Modeling Environmental Influences on Child Growth in the MAL-ED Cohort Study: Opportunities and Challenges. Clin Infect Dis. 2014;59: S255–S260. 10.1093/cid/ciu436 25305295 PMC4204605

[24] Myriad RBM. HumanMAP v. 2.0. 2018 [cited 22 Aug 2018]. Available: https://myriadrbm.com/products-services/humanmap-services/humanmap/

[25] Gray N, Zia R, King A, Patel VC, Wendon J, McPhail MJW, et al High-Speed Quantitative UPLC-MS Analysis of Multiple Amines in Human Plasma and Serum via Precolumn Derivatization with 6-Aminoquinolyl- *N* -hydroxysuccinimidyl Carbamate: Application to Acetaminophen-Induced Liver Failure. Anal Chem. 2017;89: 2478–2487. 10.1021/acs.analchem.6b04623 28194962

[26] McCormick BJJ, Lee GO, Seidman JC, Haque R, Mondal D, Quetz J, et al Dynamics and Trends in Fecal Biomarkers of Gut Function in Children from 1–24 Months in the MAL-ED Study. Am J Trop Med Hyg. 96 10.4269/ajtmh.16-0496 27994110 PMC5303054

[27] Ahmed SF, Tucker P, Mushtaq T, Wallace AM, Williams DM, Hughes IA. Short-term effects on linear growth and bone turnover in children randomized to receive prednisolone or dexamethasone. Clin Endocrinol (Oxf). 2002;57: 185–191. 10.1046/j.1365-2265.2002.01580.x 12153596

[28] Bath LE, Crofton PM, Evans AEM, Ranke MB, Elmlinger MW, Kelnar CJH, et al Bone Turnover and Growth during and after Chemotherapy in Children with Solid Tumors. Pediatr Res. 2004;55: 224–230. 10.1203/01.PDR.0000100903.83472.09 14605245

[29] Isanaka S, Kodish SR, Berthé F, Alley I, Nackers F, Hanson KE, et al Outpatient treatment of severe acute malnutrition: Response to treatment with a reduced schedule of therapeutic food distribution. Am J Clin Nutr. 2017;105: 1191–1197. 10.3945/ajcn.116.148064 28404577

[30] Hornung RW, Reed LD. Estimation of Average Concentration in the Presence of Nondetectable Values. Appl Occup Environ Hyg. 1990;5: 46–51. 10.1080/1047322X.1990.10389587

[31] Schafer JL (Joseph L. Analysis of incomplete multivariate data. Chapman & Hall; 1997 Available: https://www.crcpress.com/Analysis-of-Incomplete-Multivariate-Data/Schafer/p/book/9780412040610

[32] Storey JD, Tibshirani R. Statistical significance for genomewide studies. Proc Natl Acad Sci U S A. 2003;100: 9440–9445. 10.1073/pnas.1530509100 12883005 PMC170937

[33] Simes RJ. An improved bonferroni procedure for multiple tests of significance. Biometrika. 1986;73: 751–754. 10.1093/biomet/73.3.751

[34] StataCorp. Stata Statistical Software: Release 15. College Station, TX; 2017.

[35] Guerrant RL, Leite AM, Pinkerton R, Medeiros PHQS, Cavalcante PA, DeBoer M, et al Biomarkers of Environmental Enteropathy, Inflammation, Stunting, and Impaired Growth in Children in Northeast Brazil. PLoS One. 2016;11: e0158772 10.1371/journal.pone.0158772 27690129 PMC5045163

[36] Soliman AT, De Sanctis V, Kalra S. Anemia and growth. Indian J Endocrinol Metab. 2014;18: S1–5. 10.4103/2230-8210.145038 25538873 PMC4266864

[37] Richard SA, Mccormick BJJ, Murray-Kolb LE, Lee GO, Seidman JC, Mahfuz M, et al Enteric dysfunction and other factors associated with attained size at 5 years: MAL-ED birth cohort study findings. Am J Clin Nutr. 2019;110: 131–138. 10.1093/ajcn/nqz004 31127812 PMC6599740

[38] Hossain M, Nahar B, Haque MA, Mondal D, Mahfuz M, Naila NN, et al Serum Adipokines, Growth Factors, and Cytokines Are Independently Associated with Stunting in Bangladeshi Children. Nutrients. 2019;11 10.3390/nu11081827 31394828 PMC6723106

[39] Kamng’ona AW, Young R, Arnold CD, Kortekangas E, Patson N, Jorgensen JM, et al The association of gut microbiota characteristics in Malawian infants with growth and inflammation. Sci Rep. 2019;9: 12893 10.1038/s41598-019-49274-y 31501455 PMC6733848

[40] Bharadwaj S, Ginoya S, Tandon P, Gohel TD, Guirguis J, Vallabh H, et al Malnutrition: laboratory markers vs nutritional assessment. Gastroenterol Rep. 2016;4: 272–280. 10.1093/gastro/gow013 27174435 PMC5193064

[41] Holst JJ. The Physiology of Glucagon-like Peptide 1. Physiol Rev. 2007;87: 1409–1439. 10.1152/physrev.00034.2006 17928588

[42] Yeung EH, Sundaram R, Xie Y, Lawrence DA. Newborn adipokines and early childhood growth. Pediatr Obes. 2018;13: 505–513. 10.1111/ijpo.12283 29781193 PMC6105426

[43] Woo JG, Guerrero ML, Altaye M, Ruiz-Palacios GM, Martin LJ, Dubert-Ferrandon A, et al Human milk adiponectin is associated with infant growth in two independent cohorts. Breastfeed Med. 2009;4: 101–9. 10.1089/bfm.2008.0137 19500050 PMC2779028

[44] Semba RD, Shardell M, Sakr Ashour FA, Moaddel R, Trehan I, Maleta KM, et al Child Stunting is Associated with Low Circulating Essential Amino Acids. EBioMedicine. 2016;6: 246–252. 10.1016/j.ebiom.2016.02.030 27211567 PMC4856740

[45] Wang F-M, Lin C-M, Lien S-H, Wu L-W, Huang C-F, Chu D-M. Sex difference determined the role of sex hormone-binding globulin in obese children during short-term weight reduction program. Medicine (Baltimore). 2017;96: e6834 10.1097/MD.0000000000006834 28489766 PMC5428600

[46] Hutchinson WL, Hohenester E, Pepys MB. Human serum amyloid P component is a single uncomplexed pentamer in whole serum. Mol Med. 2000;6: 482–93. Available: http://www.ncbi.nlm.nih.gov/pubmed/10972085 10972085 PMC1949963

[47] Agrawal A, Singh PP, Bottazzi B, Garlanda C, Mantovani A. Pattern recognition by pentraxins. Adv Exp Med Biol. 2009;653: 98–116. Available: http://www.ncbi.nlm.nih.gov/pubmed/19799114 10.1007/978-1-4419-0901-5_7 19799114 PMC3367411

[48] Poulsen ET, Pedersen KW, Marzeda AM, Enghild JJ. Serum Amyloid P Component (SAP) Interactome in Human Plasma Containing Physiological Calcium Levels. Biochemistry. 2017;56: 896–902. 10.1021/acs.biochem.6b01027 28098450

[49] Tortora GJ, Derrickson BH. Principles of Anatomy and Physiology. 14th ed Wiley; 2014 Available: http://www.wiley.com/WileyCDA/WileyTitle/productCd-EHEP002935.html

[50] Kim H-Y, Mohan S. Role and Mechanisms of Actions of Thyroid Hormone on the Skeletal Development. Bone Res. 2013;1: 146–161. 10.4248/BR201302004 26273499 PMC4472099

[51] Iqbal NT, Sadiq K, Syed S, Akhund T, Umrani F, Ahmed S, et al Promising Biomarkers of Environmental Enteric Dysfunction: A Prospective Cohort study in Pakistani Children. Sci Rep. 2018;8: 2966 10.1038/s41598-018-21319-8 29445110 PMC5813024

[52] Dabrowska AM, Tarach JS, Wojtysiak-Duma B, Duma D. Fetuin-A (AHSG) and its usefulness in clinical practice. Review of the literature. Biomed Pap. 2015;159: 352–359. 10.5507/bp.2015.018 25916279

[53] Kosek MN, Lee GO, Guerrant RL, Haque R, Kang G, Ahmed T, et al Age and Sex Normalization of Intestinal Permeability Measures for the Improved Assessment of Enteropathy in Infancy and Early Childhood. J Pediatr Gastroenterol Nutr. 2017;65: 31–39. 10.1097/MPG.0000000000001610 28644347

[54] Ponka P, Beaumont C, Richardson DR. Function and regulation of transferrin and ferritin. Semin Hematol. 1998;35: 35–54. Available: http://www.ncbi.nlm.nih.gov/pubmed/9460808 9460808

[55] Taylor J, Tibshirani RJ. Statistical learning and selective inference. Proc Natl Acad Sci U S A. 2015;112: 7629–7634. 10.1073/pnas.1507583112 26100887 PMC4485109

[56] Denno DM, VanBuskirk K, Nelson ZC, Musser CA, Hay Burgess DC, Tarr PI. Use of the lactulose to mannitol ratio to evaluate childhood environmental enteric dysfunction: A systematic review. Clin Infect Dis. 2014 10.1093/cid/ciu541 25305289

