# Supplementary material for: Penalized regression models to select biomarkers of environmental enteric dysfunction associated with linear growth acquisition in a Peruvian birth cohort
Source: PLoS Negl Trop Dis. 2019 Nov 15;13(11):e0007851. doi: 10.1371/journal.pntd.0007851 (PMC6881068; doi:10.1371/journal.pntd.0007851)
Supplement: S1 List — (PDF) [file pntd.0007851.s001.pdf]

Penalized regression models to select biomarkers of environmental enteric dysfunction associated with linear growth acquisition in a Peruvian birth cohort, J. M. Colston *et al.* 2019, *PLOS Neglected Tropical Diseases*

## *S1 List: Biomarkers quantified in each panel and their units*

1. **Multiplex immunoassay panels:** Adiponectin (ug/mL); Alpha-1-Antitrypsin (AAT - mg/mL); Alpha-2-Macroglobulin (A2Macro - mg/mL); Alpha-Fetoprotein (AFP - ng/mL); Apolipoproteins (Apo) A-I (mg/mL), A-II (ng/mL), B (ug/mL), C-I (ng/mL), C-III (ug/mL), D (ug/mL), E (ug/mL), and H (ug/mL); Apolipoprotein(a) (Lp(a) - ug/mL); Beta-2-Microglobulin (B2M - ug/mL); Brain-Derived Neurotrophic Factor (BDNF - ng/mL); Cancer Antigens (CA) 125 and 19-9 (U/mL); Carcinoembryonic Antigen (CEA - ng/mL); CD 40 antigen (CD40 - ng/mL); CD40 Ligand (CD40-L - ng/mL); CD5 Antigen-like (CD5L - ng/mL); Clusterin (CLU - ug/mL); Complement C3 (C3 - mg/mL); C-Reactive Protein (CRP - ug/mL); Creatine Kinase-MB (CK-MB - ng/mL); Cystatin-C - ng/mL; EN-RAGE - ng/mL; Eotaxin-1 - pg/mL; Epithelial-Derived Neutrophil-Activating Protein 78 (ENA-78 - ng/mL); E-Selectin (ng/mL); Factor VII (ng/mL); Fatty Acid-Binding Protein, heart (FABP, heart - ng/mL); Ferritin (FRTN - ng/mL); Fetuin-A (ug/mL); Fibrinogen (mg/mL); Glucagon-like Peptide 1, active (GLP-1 active - pg/mL); Granulocyte Colony-Stimulating Factor (G-CSF - pg/mL); Granulocyte-Macrophage Colony-Stimulating Factor (GM-CSF - pg/mL); Growth Hormone (GH - ng/mL); Haptoglobin (mg/mL); Human Chorionic Gonadotropin beta (hCG - mIU/mL); Immunoglobulin A (IgA - mg/mL); Immunoglobulin E (IgE - U/mL); Immunoglobulin M (IgM - mg/mL); Insulin (uIU/mL); Intercellular Adhesion Molecule 1 (ICAM-1 - ng/mL); Interferon gamma (IFN-gamma - pg/mL); Interleukin-1 alpha (IL-1 alpha - ng/mL), beta (IL-1 beta - pg/mL) and receptor antagonist (IL-1ra - pg/mL); Interleukin (IL)-2 (pg/mL), 3 (ng/mL), 4, 5, 6, 7, 8, 10, 13 (pg/mL), 15 (ng/mL), 16, 17, 18 (pg/mL) and 23 (ng/mL); Interleukin-8 (chemokine - pg/ml); Interleukin-12 Subunit p40 (ng/mL) and p70 (pg/mL); Lectin-Like Oxidized LDL Receptor 1 (LOX-1 - ng/mL); Leptin (ng/mL); Macrophage Inflammatory Protein-1 (MIP-1) alpha and beta (pg/mL); Macrophage-Derived Chemokine (MDC - pg/mL); Matrix Metalloproteinase (MMP) 1, 3, 7, 9, 9 total and 10 (ng/mL); Monocyte Chemotactic Protein 1 (MCP-1 - pg/mL); Myeloperoxidase (MPO - ng/mL); Myoglobin (ng/mL); Neuron-Specific Enolase (NSE - ng/mL); N-terminal prohormone of brain natriuretic peptide (NT proBNP - pg/mL); Osteopontin (ng/mL); Plasminogen Activator Inhibitor 1 (PAI-1 - ng/mL); Prostate-Specific Antigen, Free (PSA-f - ng/mL); P-Selectin (ng/mL); Pulmonary and Activation-Regulated Chemokine (PARC - ng/mL); Receptor for advanced glycosylation end products (RAGE - ng/mL); Serotransferrin (Transferrin - mg/dl); Serum Amyloid P-Component (SAP - ug/mL); Sex Hormone-Binding Globulin (SHBG - nmol/L); Stem Cell Factor (SCF - pg/mL); T-Cell-Specific Protein RANTES (RANTES - ng/mL); Thrombomodulin (TM - ng/mL); Thrombospondin-1 (ng/mL); Thyroid-Stimulating Hormone (TSH - uIU/mL); Thyroxine-Binding Globulin (TBG - ug/mL); Tissue Inhibitor of Metalloproteinases 1 (TIMP-1 - ng/mL); Transthyretin (TTR - mg/dl); Tumor Necrosis Factor (TNF) alpha and beta (pg/mL); Tumor necrosis factor receptor 2 (TNFR2 - ng/mL); Vascular Cell Adhesion Molecule-1 (VCAM-1 - ng/mL); Vascular Endothelial Growth Factor (VEGF - pg/mL); Vitamin D-Binding Protein (VDBP - ug/mL); Vitamin K-Dependent Protein S (VKDPS - ug/mL); Vitronectin (ug/mL); von Willebrand Factor (vWF - ug/mL).
2. **Chemokine and proinflammatory assays**: Eotaxin-1 (pg/ml); Eotaxin-3 (pg/ml); Interleukin-8 (chemokine - pg/ml); Interferon gamma-induced protein 10 (IP-10 - pg/ml); Monocyte Chemotactic Protein (MCP) 1 and 4 (pg/ml); Macrophage-Derived Chemokine (MDC - pg/ml); Macrophage Inflammatory Protein-1 beta (MIP-1 beta - pg/ml); Thymus and activation regulated chemokine (TARC - pg/ml); Granulocyte macrophage colony-stimulating factor (GM-

CSF - pg/m); Interferon gamma (IFN-gamma - pg/ml); Interleukin (IL)-1 beta, 2, 6, 8, 10 and 12p70 (pg/ml); Tumor Necrosis Factor alpha (TNF-alpha - pg/ml).

3. **Liquid chromatography-mass spectrometry (LCMS) Oregon Analytics:** Citrulline (umol/L); Kynurenine (umol/L); Tryptophan (umol/L).
4. **LCMS Imperial College London: 1**-Methyl Nicotinamide (MNA -ng/ml); 1- and 3-methylhistidine (μM); 3-HAA - ng/ml; 3-OH-Kynurenine (ng/ml); 4-hydroxyproline (Hyp - μM); 5-OH-Indole-3-acetic Acid (5-HIAA - ng/ml); Alanine (μM); Alpha-amino-n-butyric acid (AABA - μM); Aminoadipic acid (2-aminoadipate - μM); Arginine (μM); Asparagine (μM); Aspartic acid (μM); Beta-alanine (μM); Beta-amino-iso-butyric acid (BABA - μM); Carnosine (μM); Cystathionine 282 (μM); Cystine 291 (μM); Dopamine (ng/ml); Ethanolamine (μM); Gamma-amino-n-butyric acid (GABA - μM); Glutamic acid (μM); Glutamine (μM); Glycine (μM); Histidine (μM); Homoserine (μM); Hydroxylysine - μM; Indole-3-acetic Acid (ng/ml); Isoleucine (μM); Kynurenic Acid (ng/ml); Leucine (μM); Lysine 244 (μM); Methionine (μM); NAD+ (ng/ml); Neopterin (ng/ml); Nicotinamide (ng/ml); Nicotinic Acid (ng/ml); Ornithine (μM); Phenylalanine (μM); Picolinic Acid (ng/ml); Proline (μM); Quinolinic Acid (ng/ml); Sarcosine (μM); Serine (μM); Serotonin (ng/ml); Taurine (μM); Threonine (μM); Trigonelline (ng/ml); Tyrosine (μM); Valine (μM); Xanthurenic Acid (ng/ml).
5. **MAL-ED plasma analytes:** Alpha-1-acid glycoprotein (AGP - mg/dl); Insulin-like growth factor (IGF) 1; IFG-binding protein 3 (IGFBP-3); hemoglobin (g/dL).
6. **Fecal biomarkers:** Alpha-1-antitrypsin (AAT – mg/g); Myeloperoxidase (MPO – ng/mL); Neopterin (NEO – nmol/L).
7. **Urinary biomarkers:** Lactulose concentration (umol/L); mannitol concentration (umol/L); percent lactulose recovery (%); percent mannitol recovery (%); lactulose/mannitol ratio.
