# Supplementary material for: Penalized regression models to select biomarkers of environmental enteric dysfunction associated with linear growth acquisition in a Peruvian birth cohort
Source: PLoS Negl Trop Dis. 2019 Nov 15;13(11):e0007851. doi: 10.1371/journal.pntd.0007851 (PMC6881068; doi:10.1371/journal.pntd.0007851)
Supplement: S1 Table — (PDF) [file pntd.0007851.s002.pdf]

S1 Table: Summary statistics of candidate biomarkers

	Analyte[1]	Mean	Standard Deviation			Min	Max	Obser-vations	Included in database…	
			Overall	Between	Within				7 & 15-months	7,15 & 24-months
Fecal	Alpha-I-Antitrypsin - mg/g	0.52	0.45	0.31	0.34	0.00	2.42	627	✓	✓
	Myeloperoxidase	10,656.64	11,309.88	9,218.37	8,128.48	111.11	76,479.91	636	✓	✓
	Neopterin - nmol/L	2,793.25	3,008.86	1,932.17	2,403.09	14.00	30,025.60	636	✓	✓
Urinary	Lactulose - umol/L	1.02	1.31	0.82	1.02	0.00	16.99	636	✓	✓
	Percent lactulose recovery	0.34	0.30	0.19	0.23	0.00	3.51	636	✓	✓
	Mannitol - umol/L	3.18	3.76	2.60	2.82	0.00	36.05	636	✓	✓
	Percent mannitol recovery	2.81	2.41	1.65	1.82	0.00	14.19	636	✓	✓
	Lactulose:mannitol (L/M) ratio	0.20	0.33	0.23	0.26	0.02	3.92	636	✓	✓
Plasma	1-methylhistidine - μM	7.48	8.17	5.35	5.78	7.07	170.00	398		
	1-Methylnicotinamide (MNA) - ng/ml	8.41	11.76	9.67	7.71	0.00	77.60	375	✓	✓
	3-HAA - ng/ml	35.41	66.65	65.63	24.56	0.10	466.00	140	✓	✓
	3-methylhistidine - μM	7.22	2.15	2.31	1.01	7.07	39.30	398	✓	✓
	3-OH-Kynurenine - ng/ml	35.14	65.79	69.34	12.39	5.50	730.60	143		
	4-hydroxyproline (Hyp) - μM	32.80	33.58	23.31	23.50	10.10	622.10	398	✓	✓
	5-OH-Indole-3-acetic Acid (5-HIAA) - ng/ml	10.33	17.66	16.28	10.76	0.10	221.40	437	✓	✓
	Adiponectin - ug/mL	9.52	3.88	2.88	2.72	1.80	32.00	631	✓	✓
	Alanine - μM	440.73	141.89	122.64	87.37	181.40	799.90	374	✓	✓
	alpha-1-acid glycoprotein (AGP) - mg/dl	119.11	42.39	31.34	30.81	34.00	319.00	605	✓	✓
	Alpha-1-Antitrypsin (AAT) - mg/mL	1.97	0.46	0.37	0.28	0.23	3.40	453	✓	
	Alpha-2-Macroglobulin (A2Macro) - mg/mL	2.70	1.11	0.87	0.71	0.63	15.00	452	✓	
	Alpha-amino-n-butyric acid (AABA) - μM	17.85	16.24	11.73	11.12	2.12	283.10	398	✓	✓
	Alpha-Fetoprotein (AFP) - ng/mL	14.64	10.06	10.06	0.00	1.20	39.00	20		
	Aminoadipic acid (2-aminoadipate) - μM	0.97	2.35	1.56	1.66	0.71	46.60	398		
	Apolipoprotein A-I (Apo A-I) - mg/mL	1.28	0.37	0.24	0.29	0.33	5.50	631	✓	✓

[1] All concentrations are expressed in ng/mL unless otherwise stated.

S1 Table: Summary statistics of candidate biomarkers

Analyte[1]	Mean	Standard Deviation			Min	Max	Obser-vations	Included in database…	
		Overall	Between	Within				7 & 15-months	7,15 & 24-months
Apolipoprotein A-II (Apo A-II) - ng/mL	276.12	82.91	63.41	54.63	77.00	951.00	453	✓	✓
Apolipoprotein B (Apo B) - ug/mL	756.84	272.31	235.85	170.89	201.00	2,080.00	612	✓	✓
Apolipoprotein C-I (Apo C-I) - ng/mL	248.58	97.09	73.56	62.62	43.00	1,590.00	453	✓	✓
Apolipoprotein C-III (Apo C-III) - ug/mL	149.43	119.55	68.36	95.08	20.00	2,760.00	631	✓	✓
Apolipoprotein D (Apo D) - ug/mL	47.18	14.84	11.21	10.32	12.00	129.00	612	✓	✓
Apolipoprotein E (Apo E) - ug/mL	34.66	11.83	9.10	7.99	9.80	91.00	612	✓	✓
Apolipoprotein H (Apo H) - ug/mL	191.31	63.37	44.78	46.55	58.00	991.00	631	✓	✓
Apolipoprotein(a) (Lp(a)) - ug/mL	20.46	35.38	31.64	17.00	1.70	366.00	631	✓	✓
Arginine - μM	98.37	41.24	32.84	26.63	22.70	307.20	397	✓	✓
Asparagine - μM	41.15	13.87	11.59	8.72	16.20	108.10	397	✓	✓
Aspartic acid - μM	15.01	27.52	19.13	19.02	2.90	518.50	398	✓	✓
Beta-2-Microglobulin (B2M) - ug/mL	2.98	0.78	0.67	0.44	1.20	5.90	453	✓	✓
Beta-alanine - μM	8.05	33.54	22.08	23.66	0.71	673.00	398	✓	✓
Beta-amino-iso-butyric acid (BABA) - μM	4.78	6.36	4.60	4.31	1.41	115.40	398	✓	✓
Brain-Derived Neurotrophic Factor (BDNF) - ng/mL	10.05	6.81	5.62	3.94	0.39	33.00	453	✓	✓
Cancer Antigen 125 (CA-125) - U/mL	14.89	3.78	3.78	0.00	8.00	21.00	20		
Cancer Antigen 19-9 (CA-19-9) - U/mL	10.79	6.06	6.06	0.00	1.27	22.00	20	✓	✓
Carcinoembryonic Antigen (CEA) - ng/mL	2.27	1.21	1.21	0.00	0.96	4.70	20		
Carnosine - μM	7.07	0.00	0.00	0.00	7.07	7.07	398		
CD 40 antigen (CD40) - ng/mL	1.11	0.20	0.20	0.00	0.76	1.60	20	✓	
CD40 Ligand (CD40-L) - ng/mL	1.12	1.05	1.05	0.00	0.10	3.40	20		
CD5 Antigen-like (CD5L) - ng/mL	3,401.59	1,472.36	1,287.30	803.48	869.00	13,600.00	434	✓	
Citrulline - umol/L	21.41	8.92	6.13	7.00	5.94	62.78	603	✓	✓
Clusterin (CLU) - ug/mL	176.91	39.44	39.49	5.37	76.00	313.00	189		
Complement C3 (C3) - mg/mL	1.28	0.29	0.29	0.00	0.88	2.30	20	✓	✓

S1 Table: Summary statistics of candidate biomarkers

Analyte[1]	Mean	Standard Deviation			Min	Max	Obser-vations	Included in database…	
		Overall	Between	Within				7 & 15-months	7,15 & 24-months
C-Reactive Protein (CRP) - ug/mL	4.57	11.74	7.41	8.81	0.04	117.00	631	✓	✓
Creatine Kinase-MB (CK-MB) - ng/mL	4.50	1.80	1.80	0.00	1.70	8.00	20	✓	✓
Cystathionine 282 - µM	0.75	0.29	0.19	0.21	0.71	4.40	398	✓	✓
Cystatin-C - ng/mL	947.00	129.01	127.72	21.93	666.00	1,380.00	189		
Cystine 291 - µM	7.07	0.00	0.00	0.00	7.07	7.07	398		
Dopamine - ng/ml	85.89	388.79	412.38	40.88	0.20	4,042.50	120		
EN-RAGE - ng/mL	52.59	119.86	89.75	87.28	0.27	2,780.00	631	✓	✓
Eotaxin-1 - pg/mL	633.01	323.68	249.83	223.84	0.00	2,327.86	582	✓	✓
Eotaxin-3 - pg/ml	13.86	19.31	16.42	12.19	3.96	280.79	582	✓	✓
Epithelial-Derived Neutrophil-Activating Protein 78 (ENA-78) - ng/mL	2.91	1.76	1.76	0.00	0.52	6.40	20		
E-Selectin - ng/mL	21.87	9.02	9.12	0.79	3.80	115.00	189	✓	✓
Ethanolamine - µM	7.49	9.55	6.43	6.73	2.70	182.90	398	✓	✓
Factor VII - ng/mL	484.71	138.99	112.13	84.65	19.00	982.00	453	✓	✓
Fatty Acid-Binding Protein, heart (FABP, heart) - ng/mL	6.96	15.85	8.20	11.21	4.88	221.00	189	✓	✓
Ferritin (FRTN) - ng/mL	33.26	41.24	29.58	30.63	1.80	453.00	631	✓	✓
Fetuin-A - ug/mL	833.43	238.10	201.52	129.33	122.00	1,680.00	434	✓	
Fibrinogen - mg/mL	3.53	1.71	1.20	1.31	0.03	20.00	630		
Gamma-amino-n-butyric acid (GABA) - µM	1.43	0.31	0.20	0.22	1.41	7.00	398		
Glucagon-like Peptide 1, active (GLP-1 active) - pg/mL	37.96	34.88	28.23	20.87	9.19	312.00	453	✓	✓
Glutamic acid - µM	184.07	98.85	85.05	59.16	49.20	554.80	397	✓	✓
Glutamine - µM	526.41	136.06	113.61	83.98	17.20	799.50	334	✓	
Glycine - µM	262.06	92.65	81.62	52.66	105.60	765.90	396	✓	✓
Granulocyte Colony-Stimulating Factor (G-CSF) - pg/mL	23.20	12.82	12.82	0.00	4.45	60.00	20	✓	
Granulocyte-Macrophage Colony-Stimulating Factor (GM-CSF) - pg/mL	0.39	0.30	0.17	0.24	0.18	5.16	582		

S1 Table: Summary statistics of candidate biomarkers

Analyte[1]	Mean	Standard Deviation			Min	Max	Obser-vations	Included in database…	
		Overall	Between	Within				7 & 15-months	7,15 & 24-months
Growth Hormone (GH) - ng/mL	2.71	3.95	4.07	2.00	0.16	44.00	453	✓	✓
Haptoglobin - mg/mL	1.05	1.05	0.79	0.72	0.01	6.70	631	✓	✓
Hemoglobin - g/dL	10.97	1.25	0.94	0.86	6.90	14.30	639	✓	✓
Histidine - µM	88.22	27.11	23.89	16.35	35.80	228.10	397	✓	✓
Homoserine - µM	215.74	106.71	86.66	67.93	7.07	615.40	397	✓	✓
Human Chorionic Gonadotropin beta (hCG) - mIU/mL	1.02	0.25	0.25	0.00	0.92	1.60	20	✓	
Hydroxylysine - µM	7.56	9.09	5.95	6.44	7.07	187.80	398	✓	✓
Immunoglobulin A (IgA) - mg/mL	0.71	0.33	0.27	0.20	0.07	2.50	453	✓	✓
Immunoglobulin E (IgE) - U/mL	56.83	45.76	45.76	0.00	12.73	174.00	20		
Immunoglobulin M (IgM) - mg/mL	1.98	0.83	0.66	0.53	0.06	6.90	631	✓	✓
Indole-3-acetic Acid (3-IAA) - ng/ml	448.46	526.77	444.04	337.40	25.30	3,807.40	439	✓	✓
Insulin - uIU/mL	1.79	1.09	0.93	0.62	0.15	8.30	453	✓	
Insulin-like growth factor 1 (IGF-1) - ng/ml	46.32	30.26	23.27	20.29	3.19	233.62	541	✓	✓
Insulin-like growth factor-binding protein 3 (IGFBP-3) - ng/ml	2,344.16	674.72	606.25	401.54	959.80	6,152.69	570	✓	✓
Intercellular Adhesion Molecule 1 (ICAM-1) - ng/mL	256.41	74.79	53.31	54.33	70.00	570.00	628	✓	✓
Interferon gamma (IFN-gamma) - pg/mL	5.47	8.67	8.06	5.87	0.00	79.07	582		
Interferon gamma-induced protein 10 (IP10) - pg/ml	513.74	495.60	443.06	328.27	31.44	3,914.77	582	✓	✓
Interleukin-1 alpha (IL-1 alpha) - ng/mL	0.00	0.01	0.01	0.01	0.00	0.11	453	✓	
Interleukin-1 beta (IL-1 beta) - pg/mL	1.03	3.86	2.61	2.91	0.00	46.21	582	✓	
Interleukin-1 receptor antagonist (IL-1ra) - pg/mL	213.63	275.78	210.72	180.95	48.79	2,470.00	453	✓	
Interleukin-10 (IL-10) - pg/mL	7.35	9.85	6.77	7.43	0.41	186.76	582		
Interleukin-12 Subunit p40 (IL-12p40) - ng/mL	0.55	0.15	0.12	0.09	0.17	1.10	453	✓	
Interleukin-12 Subunit p70 (IL-12p70) - pg/mL	1.02	2.03	1.33	1.55	0.00	29.93	582	✓	

S1 Table: Summary statistics of candidate biomarkers

Analyte[1]	Mean	Standard Deviation: Overall	Standard Deviation: Between	Standard Deviation: Within	Min	Max	Obser-vations	Included in database… 7 & 15-months	Included in database… 7,15 & 24-months
Interleukin-13 (IL-13) - pg/mL	4.38	0.00	0.00	0.00	4.38	4.38	20	✓	
Interleukin-15 (IL-15) - ng/mL	0.34	0.07	0.06	0.05	0.28	0.80	453	✓	
Interleukin-16 (IL-16) - pg/mL	1,030.75	1,153.70	1,153.70	0.00	396.00	5,840.00	20		
Interleukin-17 (IL-17) - pg/mL	2.94	1.57	1.21	1.00	2.05	16.00	453	✓	
Interleukin-18 (IL-18) - pg/mL	486.80	243.38	243.38	0.00	251.00	1,270.00	20		
Interleukin-2 (IL-2) - pg/mL	0.46	1.28	0.68	1.04	0.00	29.33	582	✓	
Interleukin-23 (IL-23) - ng/mL	1.55	0.56	0.44	0.36	0.48	3.30	453	✓	
Interleukin-3 (IL-3) - ng/mL	0.01	0.00	0.00	0.00	0.01	0.01	20	✓	
Interleukin-4 (IL-4) - pg/mL	19.80	0.00	0.00	0.00	19.80	19.80	20		
Interleukin-5 (IL-5) - pg/mL	7.07	0.00	0.00	0.00	7.07	7.07	20		
Interleukin-6 (IL-6) - pg/mL	2.25	3.82	3.32	2.64	0.21	53.26	582		
Interleukin-7 (IL-7) - pg/mL	8.49	0.00	0.00	0.00	8.49	8.49	20		
Interleukin-8 (IL-8 chemokine) - pg/ml	12.08	9.73	7.20	7.07	1.84	146.46	582	✓	✓
Interleukin-8 (IL-8) - pg/mL	12.95	11.71	9.15	7.71	0.00	164.31	582		
Isoleucine - µM	77.38	31.67	25.18	21.17	15.20	220.40	397	✓	✓
Kynurenic Acid (KYNA) - ng/ml	4.28	2.70	1.70	2.04	0.40	40.60	439	✓	✓
Kynurenine - umol/L	2.76	1.04	0.78	0.76	0.63	8.69	603	✓	✓
Kynurenine:Tryptophan (K/T) ratio	57.83	25.44	19.51	18.33	19.45	213.83	603	✓	✓
Lectin-Like Oxidized LDL Receptor 1 (LOX-1) - ng/mL	0.67	0.68	0.55	0.35	0.53	7.20	189	✓	✓
Leptin - ng/mL	1.23	1.45	1.23	0.99	0.06	17.00	631	✓	✓
Leucine - µM	120.34	47.80	39.12	30.94	27.50	327.50	397	✓	✓
Lysine 244 - µM	165.70	81.66	67.59	52.11	2.83	562.40	397	✓	✓
Macrophage Inflammatory Protein-1 alpha (MIP-1 alpha) - pg/mL	27.14	9.42	8.99	3.54	10.61	89.00	209		
Macrophage Inflammatory Protein-1 beta (MIP-1 beta) - pg/mL	142.20	67.11	53.95	41.65	0.00	448.78	582	✓	✓

S1 Table: Summary statistics of candidate biomarkers

Analyte[1]	Mean	Standard Deviation			Min	Max	Obser-vations	Included in database…	
		Overall	Between	Within				7 & 15-months	7,15 & 24-months
Macrophage-Derived Chemokine (MDC) - pg/mL	7,523.76	2,777.86	2,213.26	1,788.72	797.70	19,777.68	582		
Matrix Metalloproteinase-1 (MMP-1) - ng/mL	1.35	0.47	0.47	0.10	1.20	4.50	189		
Matrix Metalloproteinase-10 (MMP-10) - ng/mL	0.19	0.09	0.09	0.02	0.07	0.63	189	✓	
Matrix Metalloproteinase-3 (MMP-3) - ng/mL	2.26	1.75	1.11	1.36	0.40	16.00	631	✓	✓
Matrix Metalloproteinase-7 (MMP-7) - ng/mL	1.24	0.49	0.48	0.08	0.37	5.50	189		
Matrix Metalloproteinase-9 (MMP-9) - ng/mL	133.36	71.41	61.96	38.65	24.04	580.00	453	✓	✓
Matrix Metalloproteinase-9, total (MMP-9, total) - ng/mL	529.23	265.99	258.94	64.29	175.00	2,200.00	189		
Methionine - µM	27.60	37.97	25.16	26.96	6.40	752.80	398	✓	✓
Monocyte Chemotactic Protein 1 (MCP-1) - pg/mL	258.74	106.59	88.29	70.87	23.93	856.30	582		
Monocyte Chemotactic Protein 4 (MCP-4) - pg/ml	671.32	412.11	373.41	240.89	40.56	3,792.01	582	✓	✓
Myeloperoxidase (MPO) - ng/mL	508.04	674.54	480.59	421.05	81.32	8,530.00	209		
Myoglobin - ng/mL	22.23	57.13	32.14	46.26	2.05	1,376.00	631	✓	✓
NAD+ - ng/ml	5.60	2.57	2.33	1.41	0.20	13.00	269		
Neopterin - ng/ml	2.95	7.40	7.23	3.96	0.00	76.00	328	✓	✓
Neuron-Specific Enolase (NSE) - ng/mL	12.32	8.90	8.90	0.00	3.40	39.00	20	✓	✓
Nicotinamide - ng/ml	75.28	57.27	41.55	40.42	9.40	603.90	438	✓	✓
Nicotinic Acid - ng/ml	4.33	6.27	3.71	4.54	0.00	91.40	271		
N-terminal prohormone of brain natriuretic peptide (NT proBNP) - pg/mL	208.22	215.72	218.04	8.38	11.31	1,740.00	189		
Ornithine - µM	88.66	41.48	36.92	25.24	21.60	390.30	397	✓	✓
Osteopontin - ng/mL	98.06	57.16	56.59	12.37	18.00	226.00	189		
Phenylalanine - µM	62.62	22.35	17.72	14.66	21.90	171.60	397	✓	✓
Picolinic Acid - ng/ml	9.92	8.18	6.47	5.67	0.10	34.00	436	✓	✓
Plasminogen Activator Inhibitor 1 (PAI-1) - ng/mL	130.33	83.81	59.67	60.69	5.60	658.00	631	✓	✓

S1 Table: Summary statistics of candidate biomarkers

Analyte[1]	Mean	Standard Deviation			Min	Max	Obser-vations	Included in database…	
		Overall	Between	Within				7 & 15-months	7,15 & 24-months
Proline - µM	290.82	107.38	90.04	64.08	88.60	719.00	395	✓	✓
Prostate-Specific Antigen, Free (PSA-f) - ng/mL	0.01	0.00	0.00	0.00	0.01	0.03	20	✓	✓
P-Selectin - ng/mL	107.12	61.80	62.24	6.09	42.00	711.00	189	✓	✓
Pulmonary and Activation-Regulated Chemokine (PARC) - ng/mL	176.56	68.23	51.55	46.42	50.00	561.00	612	✓	✓
Quinolinic Acid - ng/ml	125.57	57.41	46.82	39.23	30.70	574.10	439	✓	✓
Receptor for advanced glycosylation end products (RAGE) - ng/mL	4.35	2.05	2.04	0.19	0.59	12.00	189		
Sarcosine - µM	32.62	45.54	32.68	31.24	3.50	428.00	398	✓	✓
Serine - µM	188.02	73.03	63.85	44.38	64.80	720.80	397	✓	✓
Serotonin - ng/ml	20.64	34.89	26.32	22.03	0.10	419.50	306	✓	
Serotransferrin (Transferrin) - mg/dl	312.38	91.01	71.37	56.58	65.00	1,240.00	453	✓	
Serum Amyloid P-Component (SAP) - ug/mL	7.26	2.98	2.11	2.16	2.20	21.00	631	✓	✓
Sex Hormone-Binding Globulin (SHBG) - nmol/L	118.97	56.27	43.16	38.40	5.73	332.00	631	✓	✓
Stem Cell Factor (SCF) - pg/mL	350.46	124.77	91.21	86.33	100.41	1,420.00	453	✓	✓
Taurine - µM	55.68	44.06	34.68	28.74	7.07	374.10	397	✓	✓
T-Cell-Specific Protein RANTES (RANTES) - ng/mL	47.66	30.31	23.51	20.74	0.97	260.00	631	✓	✓
Threonine - µM	122.84	54.90	46.08	34.19	27.90	499.10	397	✓	✓
Thrombomodulin (TM) - ng/mL	5.14	1.08	1.07	0.18	2.70	9.20	189		
Thrombospondin-1 - ng/mL	6,904.50	2,180.25	2,180.25	0.00	2,320.00	10,400.00	20	✓	✓
Thymus and activation regulated chemokine (TARC) - pg/ml	689.13	718.71	580.29	439.44	14.57	7,189.74	582	✓	✓
Thyroid-Stimulating Hormone (TSH) - uIU/mL	2.10	1.12	0.96	0.61	0.35	9.20	453	✓	
Thyroxine-Binding Globulin (TBG) - ug/mL	53.71	12.88	9.44	9.15	15.00	114.00	631	✓	✓
Tissue Inhibitor of Metalloproteinases 1 (TIMP-1) - ng/mL	124.92	49.40	34.70	36.38	34.00	429.00	631	✓	✓
Transthyretin (TTR) - mg/dl	14.43	4.82	3.65	3.20	3.40	72.00	453	✓	✓

S1 Table: Summary statistics of candidate biomarkers

Analyte[1]	Mean	Standard Deviation			Min	Max	Obser-vations	Included in database…	
		Overall	Between	Within				7 & 15-months	7,15 & 24-months
Trigonelline - ng/ml	9.42	11.90	9.99	7.48	0.10	104.80	293	✓	✓
Tryptophan - umol/L	50.95	15.61	11.02	11.45	4.47	110.62	603	✓	✓
Tumor Necrosis Factor alpha (TNF-alpha) - pg/mL	9.93	3.33	2.73	2.23	1.83	24.65	581	✓	✓
Tumor Necrosis Factor beta (TNF-beta) - pg/mL	20.08	3.07	3.07	0.00	19.09	30.00	20	✓	✓
Tumor necrosis factor receptor 2 (TNFR2) - ng/mL	11.15	3.91	2.69	2.90	2.50	37.00	631	✓	✓
Tyrosine - µM	74.87	28.32	22.91	17.65	18.60	186.70	397	✓	✓
Valine - µM	205.12	79.92	66.01	51.93	80.80	551.20	397	✓	✓
Vascular Cell Adhesion Molecule-1 (VCAM-1) - ng/mL	940.54	259.24	199.34	184.07	250.00	2,290.00	631	✓	✓
Vascular Endothelial Growth Factor (VEGF) - pg/mL	202.70	145.96	126.59	71.78	26.87	1,280.00	453	✓	✓
Vitamin D-Binding Protein (VDBP) - ug/mL	258.02	100.97	94.62	39.08	39.00	625.00	453	✓	✓
Vitamin K-Dependent Protein S (VKDPS) - ug/mL	10.02	2.76	2.27	1.66	2.00	25.00	434	✓	
Vitronectin - ug/mL	1,479.40	826.45	728.26	478.29	156.98	5,700.00	434	✓	
von Willebrand Factor (vWF) - ug/mL	57.83	54.59	37.90	42.10	4.10	757.00	631	✓	✓
Xanthurenic Acid - ng/ml	7.84	9.66	6.97	6.63	0.10	111.80	352	✓	
