# Supplementary material for: Penalized regression models to select biomarkers of environmental enteric dysfunction associated with linear growth acquisition in a Peruvian birth cohort
Source: PLoS Negl Trop Dis. 2019 Nov 15;13(11):e0007851. doi: 10.1371/journal.pntd.0007851 (PMC6881068; doi:10.1371/journal.pntd.0007851)
Supplement: S2 Table — (PDF) [file pntd.0007851.s003.pdf]

S2 Table: Coefficient estimates (with 95% confidence intervals) from linear regression models for biomarkers selected by SCAD along with the predicted difference in child's height 2 months after the last sample for children at the 25th and 75th percentile of the biomarker distribution adjusted for contemporaneous LAZ-score

	7 & 15 months		7, 15 & 24 months	
**Biomarker**	**Coefficient - LAZ score**	**Predicted height difference (cm) at 17 months**	**Coefficient - LAZ score**	**Predicted height difference (cm) at 26 months**
**5-OH-Indole-3-acetic Acid (5-HIAA)**	-	-	0.00 (-0.02, 0.03)	0.01
**Alpha-2-Macroglobulin (A2Macro)**	-0.11 (-0.19, -0.03)	-0.17	-	-
**Alpha-amino-n-butyric acid (AABA)**	-0.01 (-0.07, 0.05)	-0.02	-	-
**Adiponectin**	-0.01 (-0.08, 0.05)	-0.02	-0.05 (-0.12, 0.02)	-0.09
**alpha-1-acid glycoprotein (AGP)**	0.05 (-0.03, 0.13)	0.08	-0.01 (-0.08, 0.07)	-0.01
**Apolipoprotein B (Apo B)**	-	-	0.03 (-0.04, 0.10)	0.06
**Apolipoprotein C-I (Apo C-I)**	-0.10 (-0.20, -0.00)	-0.14	-	-
**Apolipoprotein D (Apo D)**	-	-	-0.02 (-0.10, 0.06)	-0.03
**Arginine**	0.04 (-0.05, 0.13)	0.08	0.09 (0.01, 0.17)	0.23
**Beta-amino-iso-butyric acid (BABA)**	-	-	-0.01 (-0.05, 0.02)	-0.08
**Citrulline**	-0.01 (-0.10, 0.08)	-0.01	0.02 (-0.05, 0.09)	0.04
**Eotaxin-3**	-	-	0.02 (-0.04, 0.07)	0.03
**Fecal Myeloperoxidase (MPO)**	0.02 (0.00, 0.05)	0.15	0.02 (-0.00, 0.04)	0.13
**Growth Hormone (GH)**	-0.00 (-0.03, 0.03)	-0.01	-	-
**Hemoglobin**	0.29 (0.08, 0.49)	0.18	0.23 (0.05, 0.42)	0.17
**Homoserine**	-0.03 (-0.06, 0.01)	-0.08	-	-
**Immunoglobulin A (IgA)**	-0.06 (-0.12, 0.01)	-0.11	-	-
**Immunoglobulin M (IgM)**	0.03 (-0.09, 0.15)	0.04	0.07 (-0.02, 0.16)	0.11
**Insulin-like growth factor-binding protein 3 (IGFBP-3)**	-	-	-0.05 (-0.12, 0.01)	-0.12
**Interleukin-8 (IL-8) chemokine**	-	-	0.03 (-0.01, 0.07)	0.12
**Lactulose**	-0.01 (-0.03, 0.01)	-0.05	-	-
**Leptin**	0.01 (-0.03, 0.06)	0.06	-0.02 (-0.06, 0.01)	-0.11
**Lysine 244**	-	-	-0.02 (-0.07, 0.03)	-0.05

S2 Table: Coefficient estimates (with 95% confidence intervals) from linear regression models for biomarkers selected by SCAD along with the predicted difference in child's height 2 months after the last sample for children at the 25th and 75th percentile of the biomarker distribution adjusted for contemporaneous LAZ-score

	7 & 15 months		7, 15 & 24 months	
**Biomarker**	**Coefficient - LAZ score**	**Predicted height difference (cm) at 17 months**	**Coefficient - LAZ score**	**Predicted height difference (cm) at 26 months**
**Monocyte Chemotactic Protein 4 (MCP-4)**	-	-	-0.01 (-0.07, 0.05)	-0.04
**Myoglobin**	-	-	0.01 (-0.03, 0.06)	0.04
**Pulmonary and Activation-Regulated Chemokine (PARC)**	-0.02 (-0.09, 0.05)	-0.03	-0.04 (-0.11, 0.03)	-0.07
**Proline**	-0.08 (-0.18, 0.01)	-0.17	-0.11 (-0.18, -0.03)	-0.21
**Serum Amyloid P-Component (SAP)**	-0.06 (-0.13, 0.01)	-0.13	-0.02 (-0.09, 0.06)	-0.05
**Sarcosine**	0.04 (0.00, 0.08)	0.19	-	-
**Sex Hormone-Binding Globulin (SHBG)**	-0.02 (-0.07, 0.03)	-0.06	-0.04 (-0.09, 0.01)	-0.14
**Thymus and activation regulated chemokine (TARC)**	-	-	0.01 (-0.03, 0.06)	0.06
**Thyroxine-Binding Globulin (TBG)**	-	-	0.04 (-0.08, 0.15)	0.04
**Transferrin**	0.14 (0.01, 0.27)	0.19	-	-
**Tryptophan**	0.09 (0.00, 0.17)	0.15	0.04 (-0.02, 0.10)	0.08
**Thyroid-Stimulating Hormone (TSH)**	-0.06 (-0.11, -0.02)	-0.15	-	-
**von Willebrand Factor (vWF)**	-0.01 (-0.05, 0.03)	-0.04	-	-
