# Supplementary material for: Penalized regression models to select biomarkers of environmental enteric dysfunction associated with linear growth acquisition in a Peruvian birth cohort
Source: PLoS Negl Trop Dis. 2019 Nov 15;13(11):e0007851. doi: 10.1371/journal.pntd.0007851 (PMC6881068; doi:10.1371/journal.pntd.0007851)
Supplement: S3 Table — (PDF) [file pntd.0007851.s004.pdf]

Penalized regression models to select biomarkers of environmental enteric dysfunction associated with linear growth acquisition in a Peruvian birth cohort, J. M. Colston *et al.* 2019, *PLOS Neglected Tropical Diseases*

S3 Table: STROBE Checklist

	Item No	Recommendation
**Title and abstract**	1	(*a*) Indicate the study's design with a commonly used term in the title or the abstract – title and abstract indicate that this was a birth cohort study design
		(*b*) Provide in the abstract an informative and balanced summary of what was done and what was found – see abstract
**Introduction**		
Background/rationale	2	Explain the scientific background and rationale for the investigation being reported – Background, paragraphs 1 – 3.
Objectives	3	State specific objectives, including any prespecified hypotheses – Background, paragraph 4.
**Methods**		
Study design	4	Present key elements of study design early in the paper – Methods paragraphs 1 – 3.
Setting	5	Describe the setting, locations, and relevant dates, including periods of recruitment, exposure, follow-up, and data collection – Methods, paragraphs 1 - 3
Participants	6	(*a*) Give the eligibility criteria, and the sources and methods of selection of participants. Describe methods of follow-up – Methods, paragraphs 1 and 2.
		(*b*) For matched studies, give matching criteria and number of exposed and unexposed – Not applicable
Variables	7	Clearly define all outcomes, exposures, predictors, potential confounders, and effect modifiers. Give diagnostic criteria, if applicable – Methods paragraphs 2 and 3.
Data sources/ measurement	8*	For each variable of interest, give sources of data and details of methods of assessment (measurement). Describe comparability of assessment methods if there is more than one group – Methods paragraphs 2 and 3.
Bias	9	Describe any efforts to address potential sources of bias – Not applicable.
Study size	10	Explain how the study size was arrived at – previous publications in which this is explained are cited in Methods, paragraph 1.
Quantitative variables	11	Explain how quantitative variables were handled in the analyses. If applicable, describe which groupings were chosen and why – Methods, statistical analysis section.

Statistical methods	12	(*a*) Describe all statistical methods, including those used to control for confounding – Methods, statistical analysis section.
		(*b*) Describe any methods used to examine subgroups and interactions – Not applicable
		(*c*) Explain how missing data were addressed – Methods, statistical analysis section.
		(*d*) If applicable, explain how loss to follow-up was addressed – Not applicable
		(*e*) Describe any sensitivity analyses – Methods, final paragraph. The same statistical methods were applied to two versions of the data (the 7–15 month and the 7-24 months databases) as a sensitivity analysis.
**Results**		
Participants	13*	(a) Report numbers of individuals at each stage of study—eg numbers potentially eligible, examined for eligibility, confirmed eligible, included in the study, completing follow-up, and analysed – Results, paragraph 1 and Supplementary Figure S1
		(b) Give reasons for non-participation at each stage - Supplementary Figure S1
		(c) Consider use of a flow diagram - Supplementary Figure S1
Descriptive data	14*	(a) Give characteristics of study participants (eg demographic, clinical, social) and information on exposures and potential confounders – supplementary table S1.
		(b) Indicate number of participants with missing data for each variable of interest – supplementary table S1.
		(c) Summarise follow-up time (eg, average and total amount) – not applicable, since no incidence rates are reported.
Outcome data	15*	Report numbers of outcome events or summary measures over time – not applicable
Main results	16	(*a*) Give unadjusted estimates and, if applicable, confounder-adjusted estimates and their precision (eg, 95% confidence interval). Make clear which confounders were adjusted for and why they were included – unadjusted estimates were not relevant to this study. Confounder adjusted estimates are given in figure 1 and confounders are listed in the Methods section
		(*b*) Report category boundaries when continuous variables were categorized – not applicable
		(*c*) If relevant, consider translating estimates of relative risk into absolute risk for a meaningful time period – not relevant.

Other analyses	17	Report other analyses done—eg analyses of subgroups and interactions, and sensitivity analyses – not applicable.
**Discussion**		
Key results	18	Summarise key results with reference to study objectives – Discussion, paragraph 1.
Limitations	19	Discuss limitations of the study, taking into account sources of potential bias or imprecision. Discuss both direction and magnitude of any potential bias – limitations are mentioned in Discussion paragraph 8. Potential bias was not considered relevant.
Interpretation	20	Give a cautious overall interpretation of results considering objectives, limitations, multiplicity of analyses, results from similar studies, and other relevant evidence Discussion, paragraphs 1 – 7.
Generalisability	21	Discuss the generalisability (external validity) of the study results – Discussion, paragraph 7.
**Other information**		
Funding	22	Give the source of funding and the role of the funders for the present study and, if applicable, for the original study on which the present article is based – Funding sources.
