# Supplementary figures and images for: Penalized regression models to select biomarkers of environmental enteric dysfunction associated with linear growth acquisition in a Peruvian birth cohort
Source: PLoS Negl Trop Dis. 2019 Nov 15;13(11):e0007851. doi: 10.1371/journal.pntd.0007851 (PMC6881068; doi:10.1371/journal.pntd.0007851)

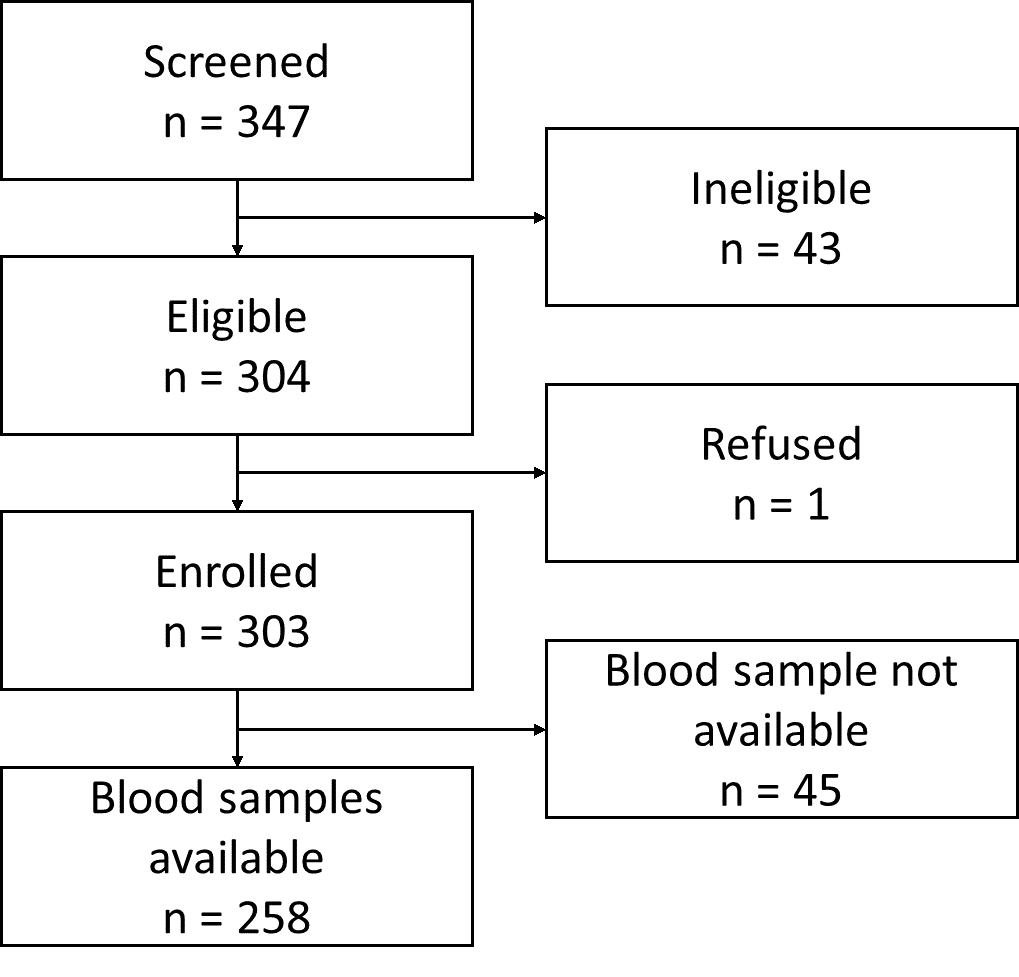

Supplement: S1 Fig — (TIF) [file pntd.0007851.s005.tif]

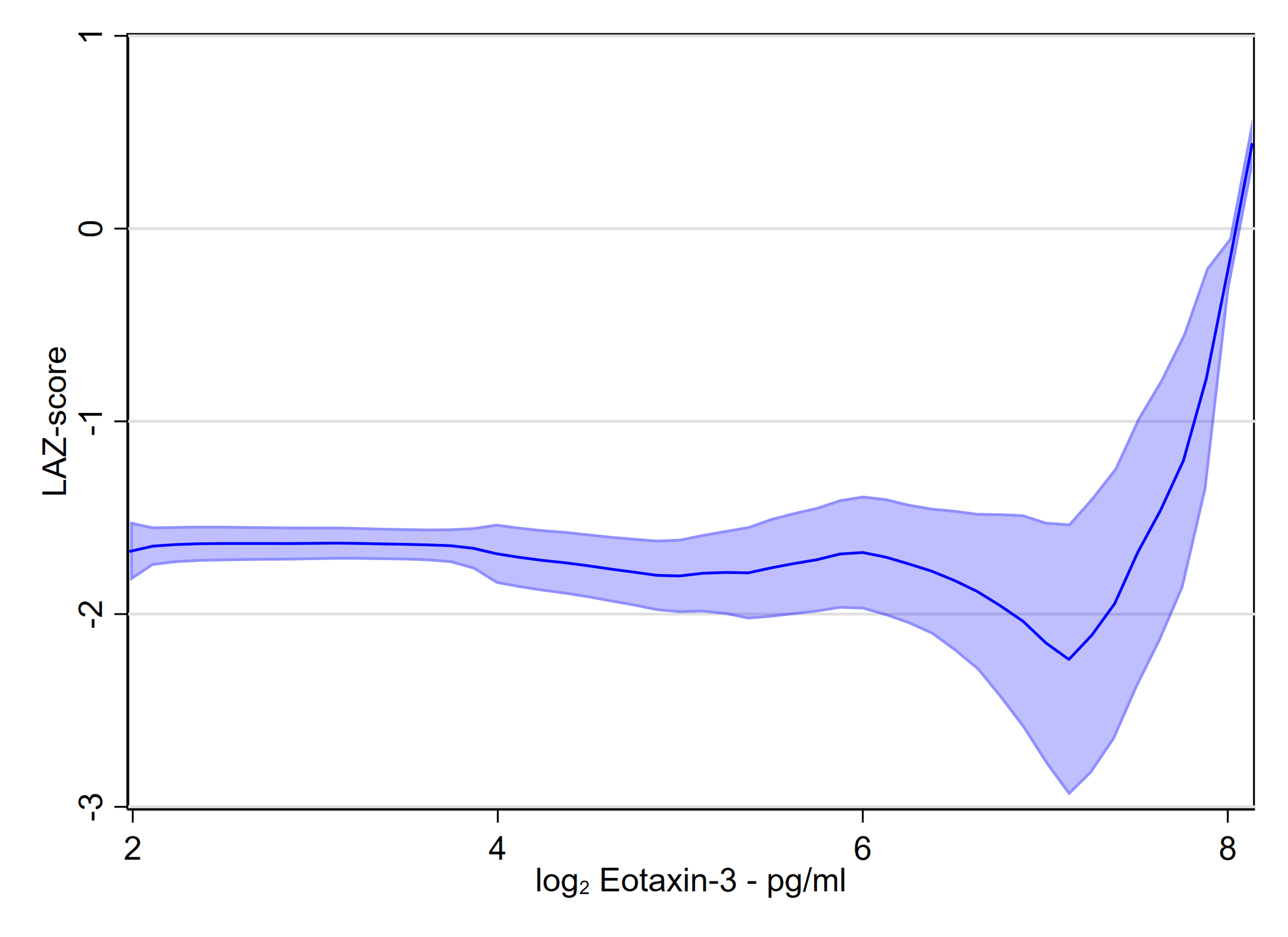

Supplement: S2 Fig — (TIF) [file pntd.0007851.s006.tif]

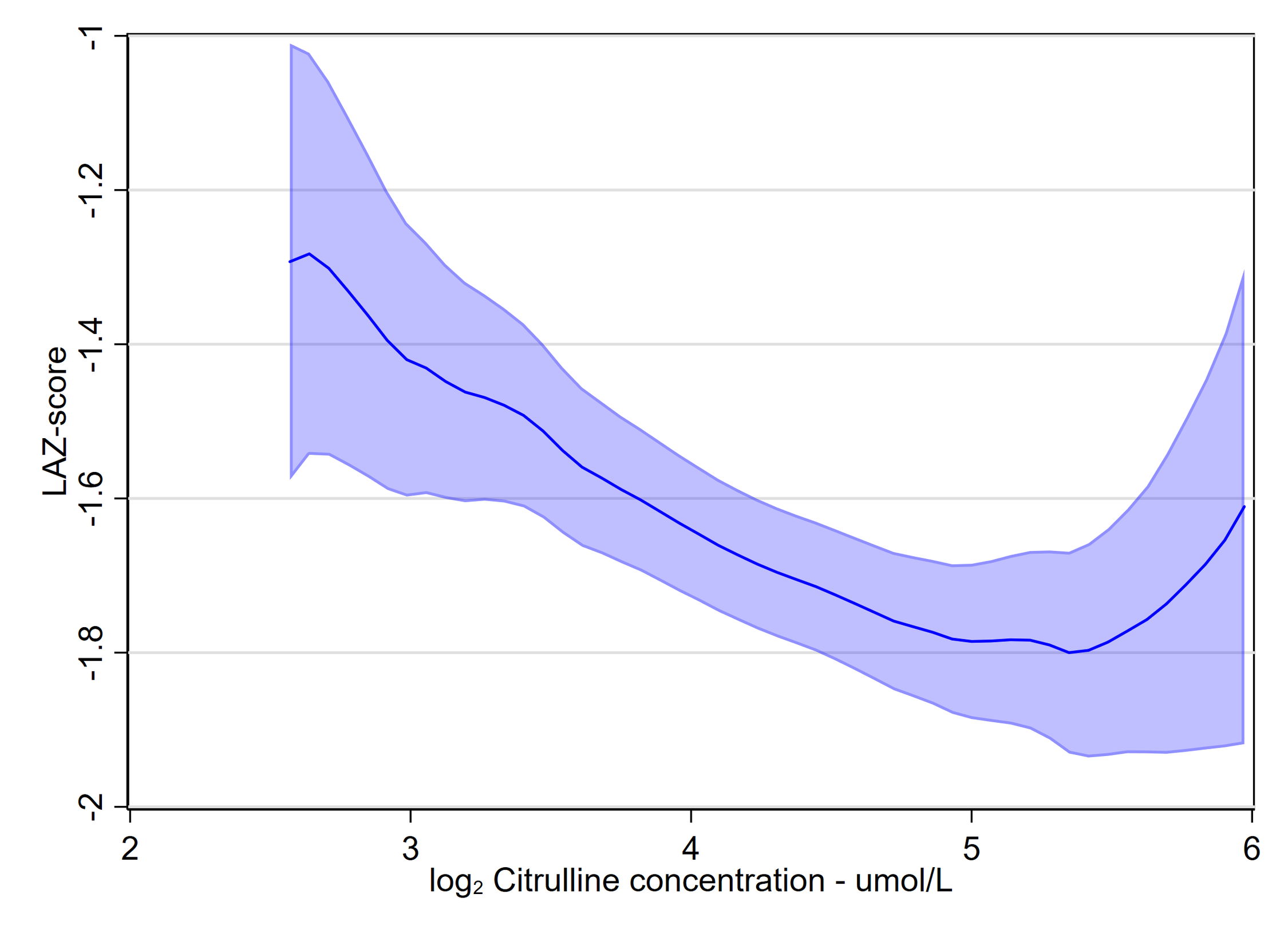

Supplement: S3 Fig — (TIF) [file pntd.0007851.s007.tif]

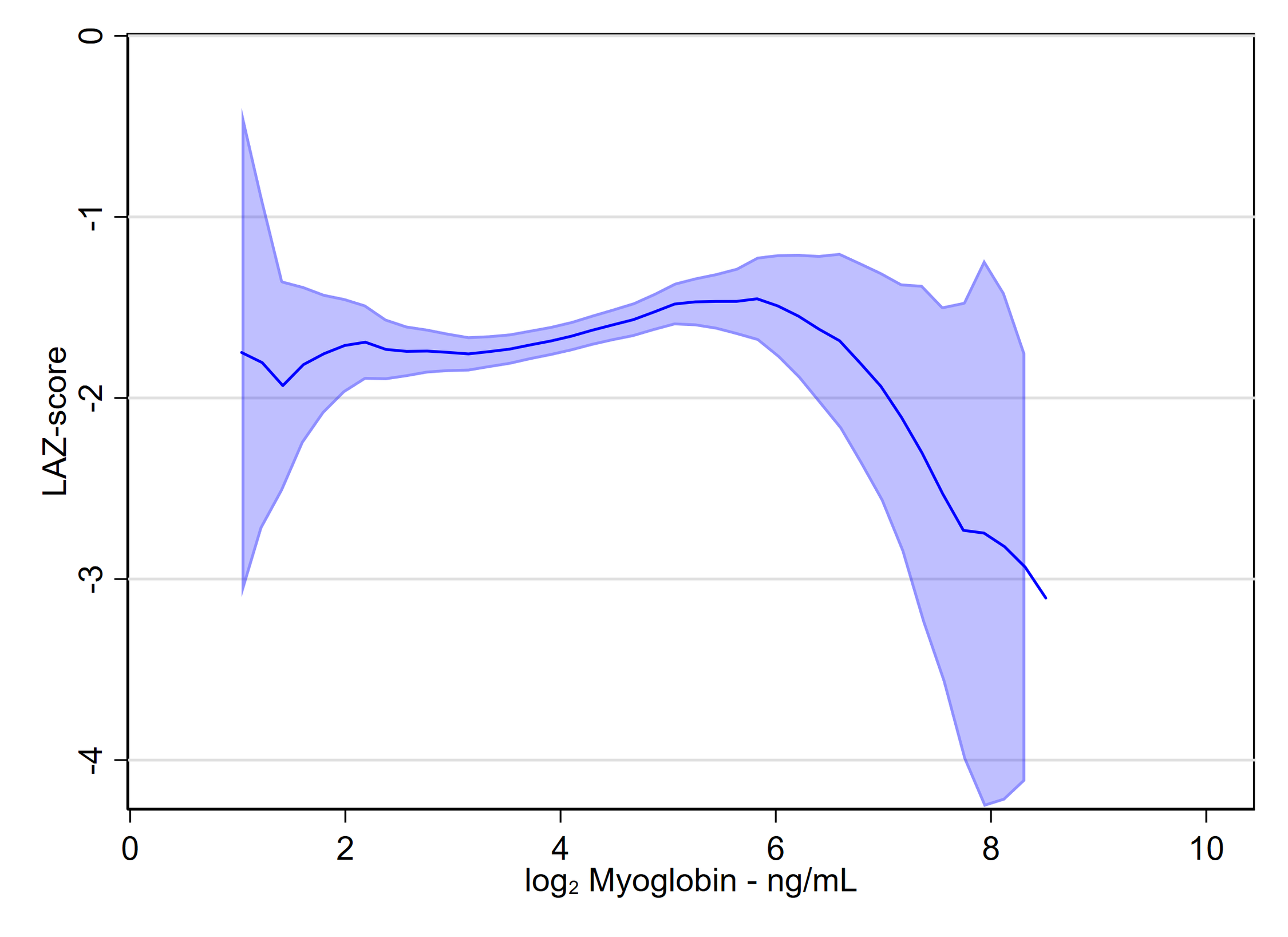

Supplement: S4 Fig — (TIF) [file pntd.0007851.s008.tif]

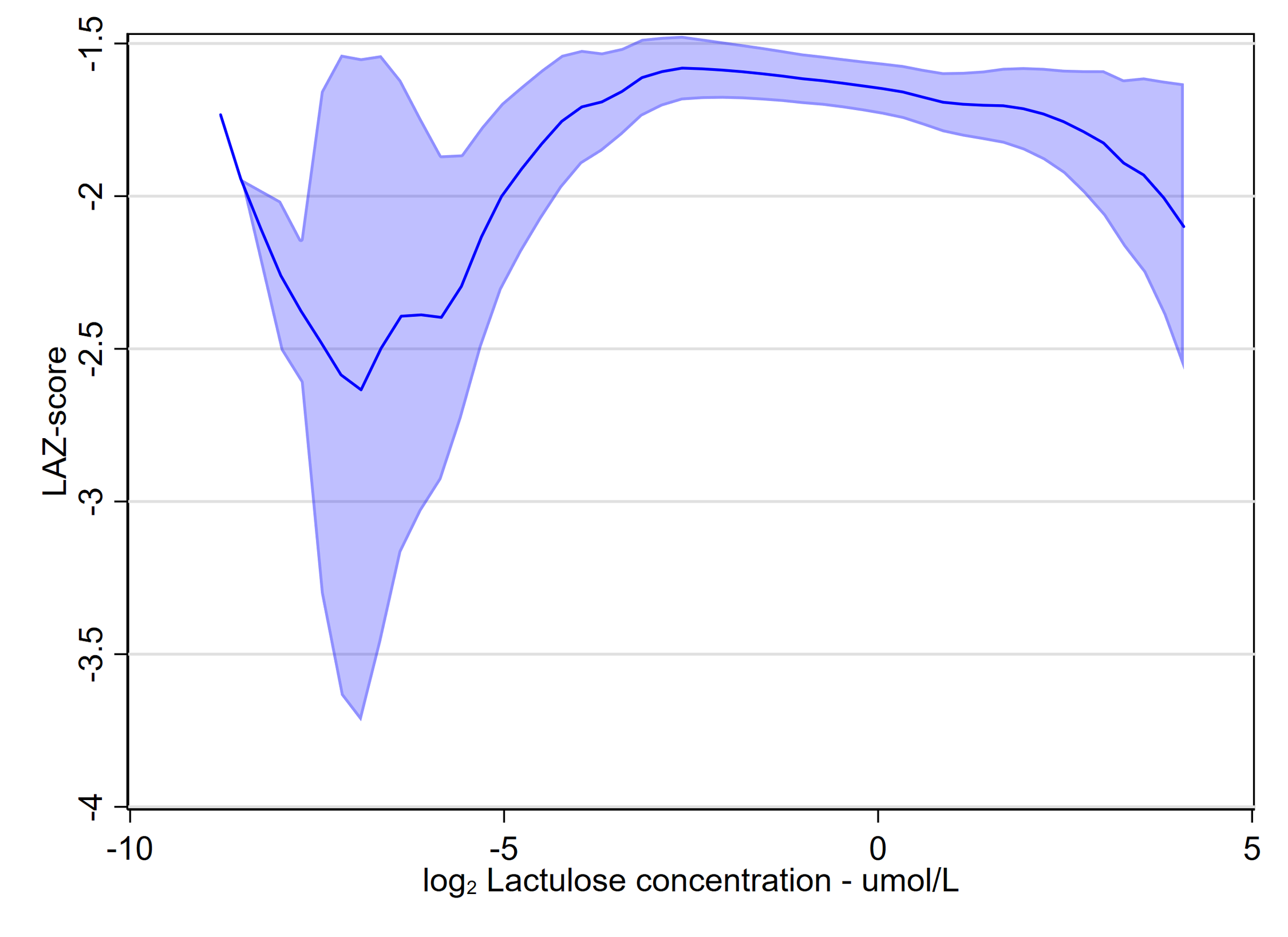

Supplement: S5 Fig — (TIF) [file pntd.0007851.s009.tif]

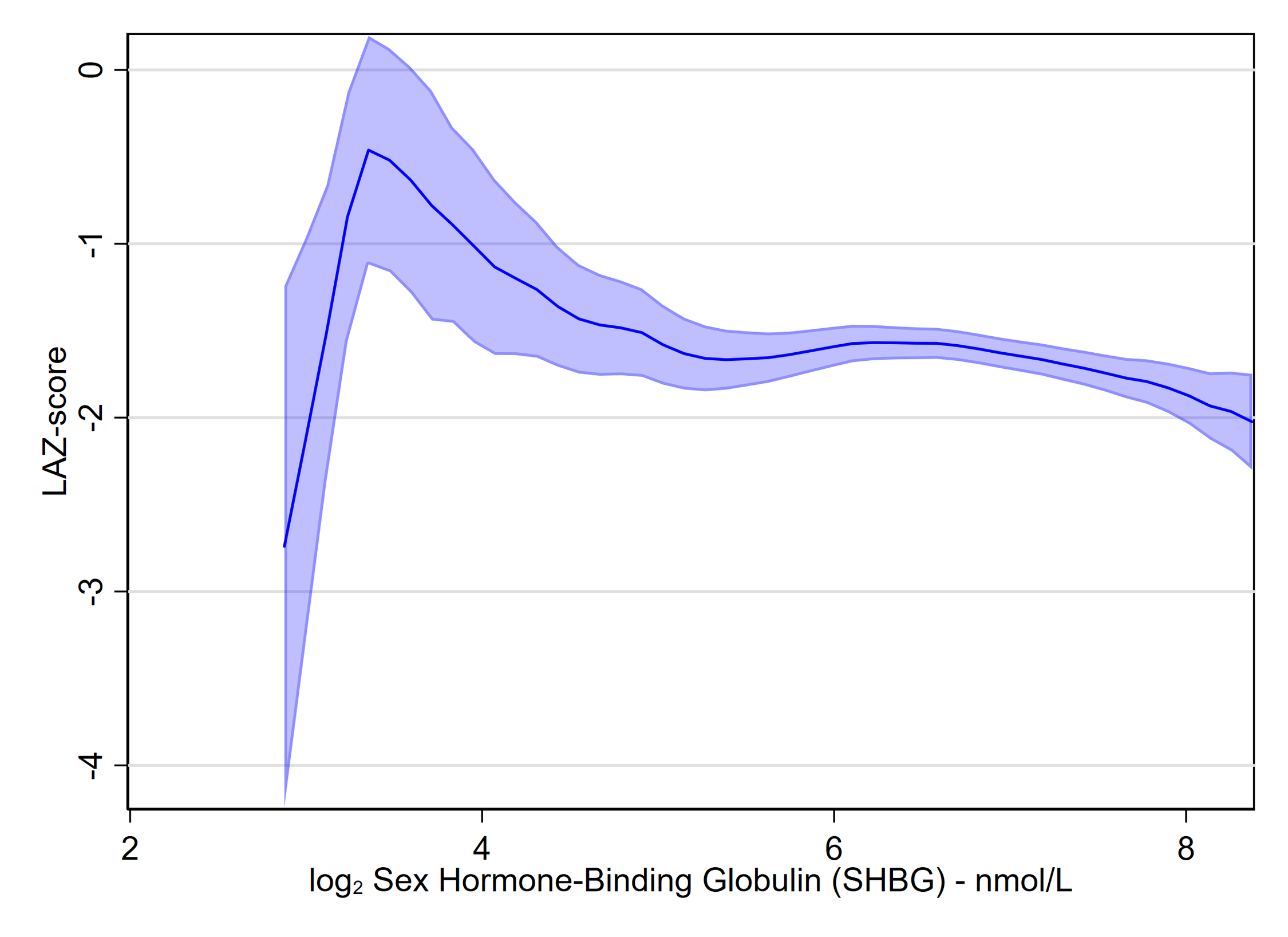

Supplement: S6 Fig — (TIF) [file pntd.0007851.s010.tif]
